# Relationship of the CreBC two-component regulatory system and inner membrane protein CreD with swimming motility in *Stenotrophomonas maltophilia*

**DOI:** 10.1371/journal.pone.0174704

**Published:** 2017-04-24

**Authors:** Hsin-Hui Huang, Wei-Ching Chen, Cheng-Wen Lin, Yi-Tsung Lin, Hsiao-Chen Ning, Yi-Chih Chang, Tsuey-Ching Yang

**Affiliations:** 1 Department of Biotechnology and Laboratory Science in Medicine, National Yang-Ming University, Taipei, Taiwan; 2 Super Laboratory Co. Ltd., New Taipei City, Taiwan; 3 Department of Medical Laboratory Science and Biotechnology, China Medical University, Taichung, Taiwan; 4 Division of Infectious Diseases, Department of Medicine, Taipei Veterans General Hospital, Taipei, Taiwan; 5 School of Medicine, National Yang-Ming University, Taipei, Taiwan; 6 Department of Laboratory Medicine, Chang Gung Memorial Hospital Linkou Branch, Taoyuan, Taiwan; 7 Department of Medical Biotechnology and Laboratory Science, Chang Gung University, Taoyuan, Taiwan; Centre National de la Recherche Scientifique, Aix-Marseille Université, FRANCE

## Abstract

The CreBC two-component system (TCS) is a conserved regulatory system found in *Escherichia coli*, *Aeromonas* spp., *Pseudomonas aeruginosa*, and *Stenotrophomonas maltophilia*. In this study, we determined how CreBC TCS regulates secreted protease activities and swimming motility using *creB*, *creC*, and *creBC* in-frame deletion mutants (KJΔCreB, KJΔCreC, and KJΔBC) of *S*. *maltophilia* KJ. Compared to wild-type KJ, KJΔCreB had a comparable secreted protease activity; however, the secreted protease activities were obviously reduced in KJΔCreC and KJΔBC, suggesting that CreC works together with another unidentified response regulator (not CreB) to regulate secreted protease activity. Single gene inactivation of *creB* or *creC* resulted in mutants with an enhanced swimming motility, and this phenotype was exacerbated in a double mutant KJΔBC. To elucidate the underlying mechanism responsible for the *ΔcreBC*-mediated swimming enhancement, flagella morphology observation, RNA-seq based transcriptome assay, qRT-PCR, and membrane integrity and potential assessment were performed. Flagella morphological observation ruled out the possibility that swimming enhancement was due to altered flagella morphology. *CreBC* inactivation upregulated the expression of *creD* and flagella-associated genes encoding the basal body- and motor-associated proteins. Furthermore, KJΔBC had an increased membrane susceptibility to Triton X-100 and CreD upregulation in KJΔBC partially alleviated the compromise of membrane integrity. The impact of *creBC* TCS on bacterial membrane potential was assessed by carbonyl cyanide m-chlorophenyl hydrazine (CCCP_50_) concentration at which 50% of bacterial swimming is inhibited. CCCP_50_ of wild-type KJ increased when *creBC* was deleted, indicating an association between the higher membrane potential of KJΔBC cells and enhanced motility. Upregulation of the basal body- and motor-associated genes of flagella in KJΔBC cells may explain the increased membrane potential. Collectively, inactivation of *creBC* increased swimming motility through membrane potential increase and *creD* upregulation in *S*. *maltophilia*. The increased membrane potential may supply more energy for flagella propelling and CreD upregulation supports membrane stability, providing a strong membrane for flagellum function.

## Introduction

*Stenotrophomonas maltophilia* is both a commensal microbe and opportunistic human pathogen that occurs naturally in a variety of habitats [1]. The ubiquitous nature of this microorganism stems mostly from its capacity to survive a variety of environmental conditions with the aid of its stress defense mechanisms. Two-component regulatory systems (TCSs) constitute a critical set of regulators that sense environmental signals and respond by coordinating the expression of an array of genes [2]. TCSs are composed of an inner membrane sensor kinase (SK), acting as a signal sensor, and a cognate response regulator (RR), which works as a transcription factor to activate or repress the expression of a variety of genes of the TCS regulon [3]. The genome of *S*. *maltophilia* K279a is equipped with at least 43 sets of TCSs [4], but only a few have been characterized, including SmeSR, SmeRySy, BfmAK, and CreBC. The SmeSR and SmeRySy systems are involved in the regulation of RND-type efflux pumps, as well as in multidrug resistance [5–6]. The BfmAK system controls biofilm development [7].

CreBC/BlrAB is a conserved TCS in many gram-negative bacteria such as *Escherichia coli*, *Aeromonas* spp. (named as BlrAB), *Pseudomonas aeruginosa*, and *S*. *maltophilia*. CreC/BlrB functions as an SK and CreB/BlrA as an RR. The functions of CreBC/BlrAB TCSs in *E*. *coli*, *Aeromonas* spp., and *P*. *aeruginosa* can be discerned when these systems are activated [8–10]. The CreBC TCS of *E*. *coli* is responsive to carbon sources and oxygen availability, and its activation is beneficial, as it mediates growth adaption to anaerobic environments [8]. The BlrAB TCS of *Aeromonas* spp., which acts as a regulator for β-lactamase expression, is activated by β-lactam challenge or by the functional loss of penicillin-binding protein 4 (PBP4) [10–12]. In response to PBP4 inactivation, the activated CreBC TCS of *P*. *aeruginosa* plays a major role in fitness, biofilm growth, and global regulation [13]. In addition, components of the *creBC*/*blrAB* regulon in *E*. *coli*, *Aeromonas* spp., and *P*. *aeruginosa* have also reported. Among these, a tightly controlled *cre* regulon gene was reported, namely *creD/blrD*, which is located downstream of the *creBC*/*blrAB* operon and is highly conserved in CreBC/BlrAB-harboring microorganisms. *CreD* expression is upregulated by activation of CreBC in *E*. *coli*, *Aeromonas* spp., and *P*. *aeruginosa* [8–9]; therefore, *creD* upregulation is considered an indicator of *creBC* TCS activation in systems of these bacterial species. Compared to those of *E*. *coli*, *Aeromonas* spp. and *P*. *aeruginosa*, the CreBC TCS of *S*. *maltophilia* has some unique features. The *creBC* operon of *S*. *maltophilia* is constitutively transcribed under laboratory culture conditions [14], although the extracellular stimulating signals remains unknown. There may be signals that further stimulate the *creBC* system. Furthermore, in contrast to *E*. *coli*, *Aeromonas* spp., and *P*. *aeruginosa*, *creD* of *S*. *maltophilia* is expressed separately from the adjacent *creBC* operon and has its own promoter. The promoter of *creD* (*P*_*creD*_) is negatively regulated by *creBC* and positively regulated by bacterial culture density. Factors compromising bacterial growth such as plasmid carriage or antibiotics attenuate the promoter activity of *P*_*creD*_ [14]. CreD of *S*. *maltophilia* is responsible for cell division and cell envelope integrity [14].

Bacteria have developed different motility systems to move, ensuring a survival advantage under a wide variety of environments. Flagella-based swimming motility is a major mode of locomotion for bacterial movement through liquids. The bacterial flagellum is composed of approximately 20 proteins, with approximately 30 additional proteins required for its regulation and assembly [15]. The flagellum is usually described in three parts, specifically the basal body, the hook, and the helical filament [16]. The basal body is embedded within the cell membrane and is composed of a rotor, stator, and periplasmic rod. The rotor includes the cytoplasmic membrane MS ring (FliN protein) and the cytoplasmic C ring (FliG, FliM, and FliN proteins), which acts as a switch to determine the rotation of flagellum. MotA and MotB proteins are the main components of the stator. The proton flow from the periplasm to the cytoplasm mediated by the stator complex MotA/MotB is coupled to the rotation of the flagellum and thus drives swimming [17]. The periplasmic rod (FliE, FlgB, FlgC, FlgF, and FlgG proteins) runs between the hook and basal body, passing through the peptidoglycan layer-P ring (FlgI protein) and outer membrane-L ring (FlgH protein). The hook, connecting the basal body to the filament, consists of FlgE, FlgK, and FlgL. The helical filament, which is composed of flagellin (FliC) and the distal FilD cap, functions as a propeller [16].

Little is known about the function of CreBC in *S*. *maltophilia*, except that inactivation of MltD1 (a lytic transglycosylase) elicits a CreBC-mediated elevation in β-lactamase activity in the absence of β-lactam [18]. Since the CreBC TCS is active in laboratory culture conditions, without stress challenge, this prompted us to consider the involvement of the CreBC TCS of *S*. *maltophilia* in bacterial physiology. In this study, we sought to define the function of CreBC in bacterial growth, morphology, secreted protease activity, and swimming motility. Based on phenotypic and genetic studies of *creB*, *creC*, and *creBC* mutants, we demonstrated that CreC likely modulates swimming motility via the response regulator CreB, whereas CreC might be involved in cross talk with another unidentified response regulator, to modulate secreted protease activity. Furthermore, inactivation of *creBC* increases inner membrane potential and upregulates *creD*, which can contribute to enhanced swimming motility.

## Results

### Growth characteristics and morphology of the *creBC* mutant

KJΔBC, an isogenic *creBC* in-frame deletion mutant, was constructed in our recent study [14]. The growth of KJ and KJΔBC was assessed by monitoring the OD_450nm_ every 3 h. These strains exhibited indistinguishable growth patterns at 37°C (data not shown).

**Fig 1:**
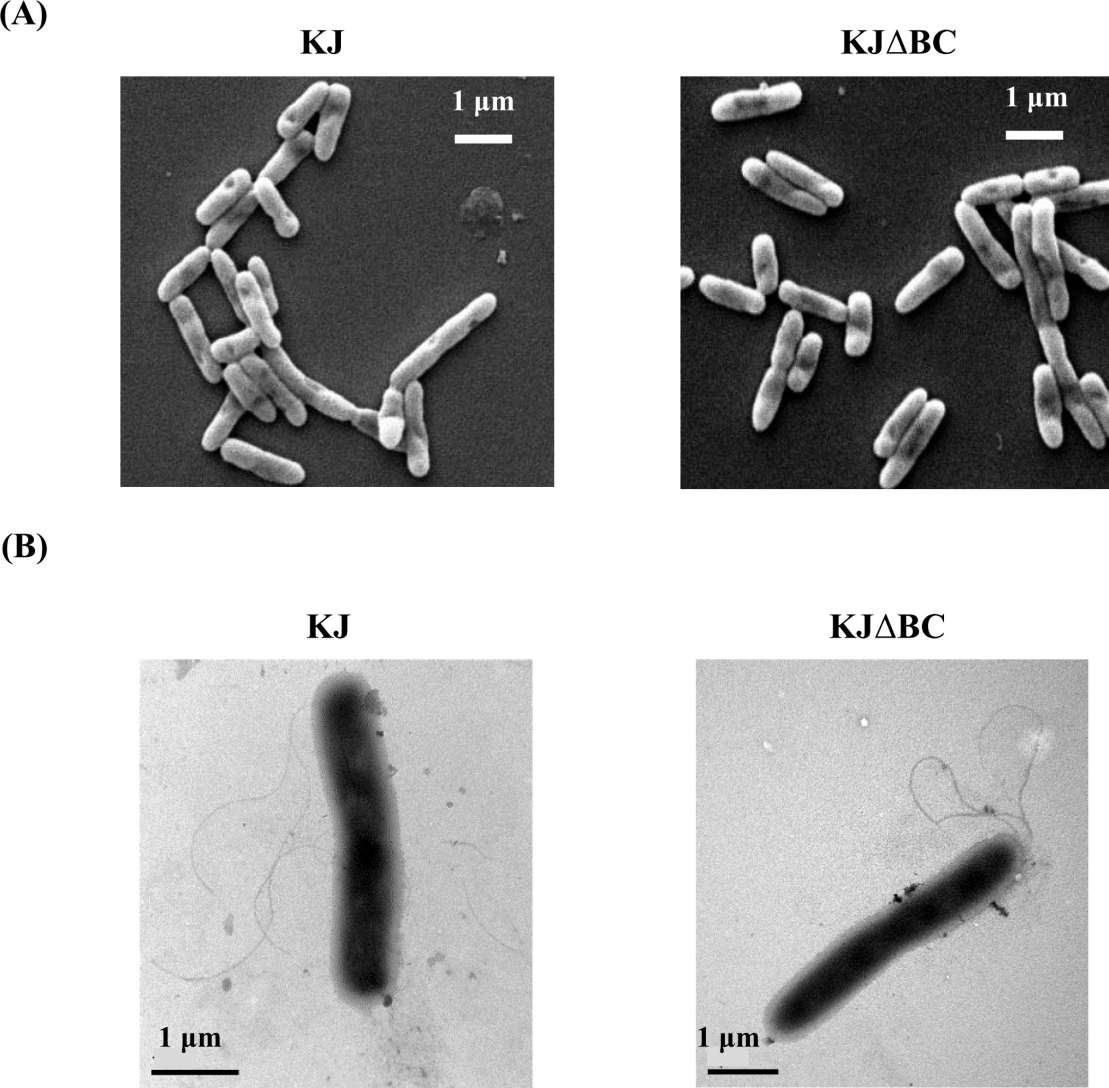
Role of CreBC TCS in bacterial and flagella morphologies. The overnight-cultured bacteria were adjusted to an initial OD_450_ of 0.15. After a 5-h incubation at 37°C, the logarithmic-phase cells were harvested for morphology observation. (A) Bacterial morphology. The samples for bacterial morphology observation were processed by glutaraldehyde-osmium tetraoxide (OsO_4_)-ethanol method and examined suing SEM. (B) Flagella morphology. The flagella were negatively strained with 1% phosphotungstic acid (pH 7.4) and observed by TEM.

The impact of *creBC* inactivation on bacterial morphology was assessed by SEM. No observable morphological aberrations were noticed when comparing KJ and KJΔBC strains (Fig 1A).

### *CreC* and *creBC* mutants, but not a *creB* mutant, display decreased secreted protease activity

**Fig 2:**
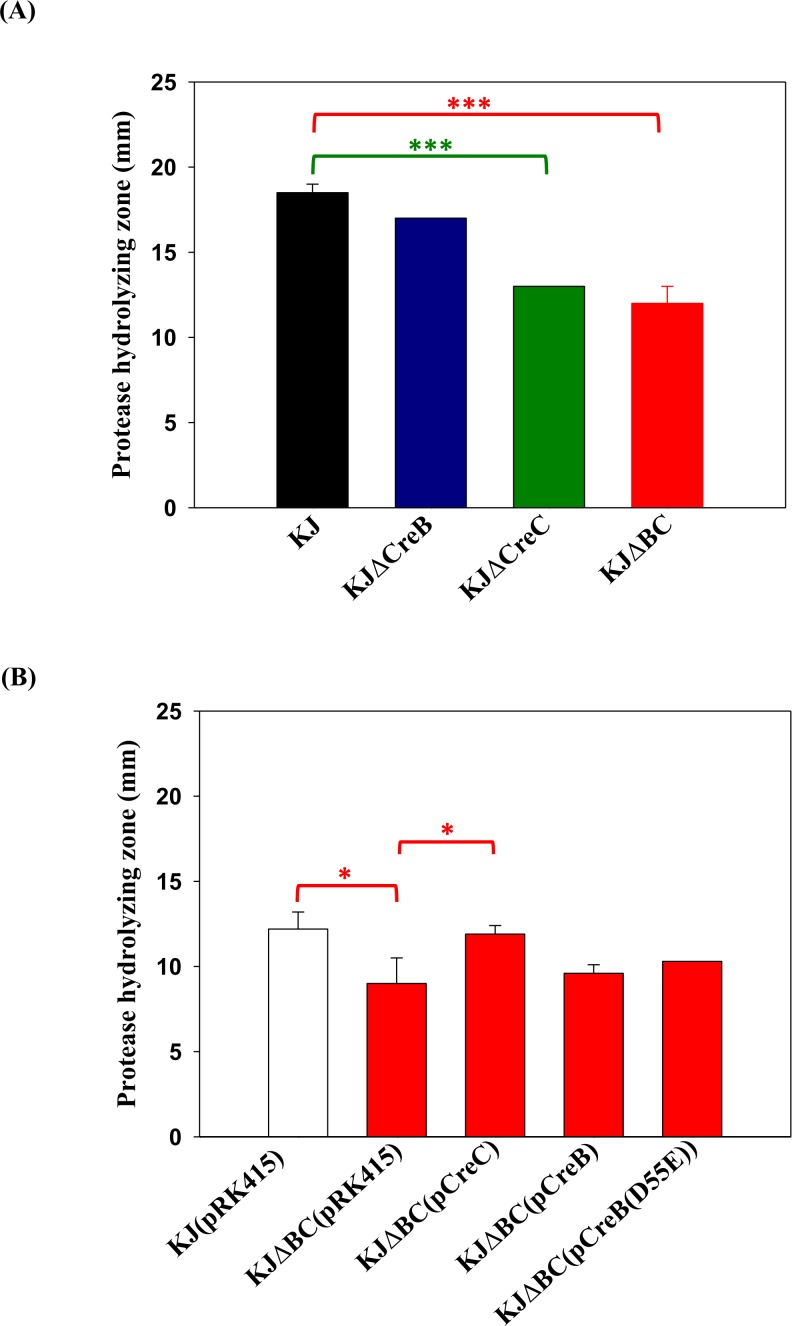
The role of CreBC in the secreted protease activity. Forty microliters of bacterial cell suspension was dipped onto LB agar containing 1% skim milk. After incubation at 37°C for 72 hour, the proteolytic activity of bacteria was assessed by measuring the transparent zones around the bacteria. Data represent the means from 3 independent experiments. Error bars represent the standard deviations for three triplicate samples. *, *p* < 0.05; ***, *p* < 0.001. (A) The secreted protease activities of *creB*, *creC*, and *creBC* mutants. (B) The secreted protease activities of *creBC* mutant and its derived complementation strains. Tetracycline (30 μg/ml) was added for the plasmid maintenance.

The activity of protease secreted from the bacteria was assayed using LB agar containing 1% skim milk. The protease hydrolyzing zones of KJΔCreC and KJΔBC were smaller than that of wild-type KJ; whereas, KJΔCreB displayed secreted protease activity that was similar to that of wild-type KJ (Fig 2A). This observation prompted us to consider the possibility that CreC is cognate with an unidentified response regulator, and governs secreted protease activity. To test this possibility, *creC* complementation assay was performed. We noticed that the hydrolysing zones observed in plasmid-carrying strains were generally smaller than those of their deletion-mutant counterparts [for example KJ vs. KJ(pRK415) and KJΔBC vs. KJΔBC(pRK415)] (Fig 2B). This might have resulted from the addition of tetracycline for plasmid maintenance. The protease hydrolysing zones of KJΔBC was reverted to the wild-type level when intact *creC* was complemented (KJΔBC(pCreC) in Fig 2B). To further verify the irrelevance of CreB in secreted protease activity, a complementation assay was performed. We used two different strategies to mimic constitutive activation of CreB to enable analysis of the corresponding CreB signalling pathway; one involved overexpression of CreB, while the other was overexpression of CreB(D55E) [14], in which amino acid 55 in CreB was converted from aspartate to glutamate. It is widely accepted that a mutation converting the conserved aspartate to glutamate at the site of phosphorylation constitutively activates the response regulator, acting as a phosphor-mimic variant of the response regulator. The plasmids pCreB and pCreB(D55E) did not rescue the effect of *creBC* deletion on secreted protease activity [KJΔBC(pRK415) vs. KJΔBC(pCreB) and KJΔBC(pCreB(D55E)] (Fig 2B), further confirming that CreB is not involved in secreted protease activity. These results supported the possibility that the CreC SK works together with another unidentified response regulator (not CreB) to regulate secreted protease activity.

### The *creBC* mutant displays enhanced swimming motility

**Fig 3:**
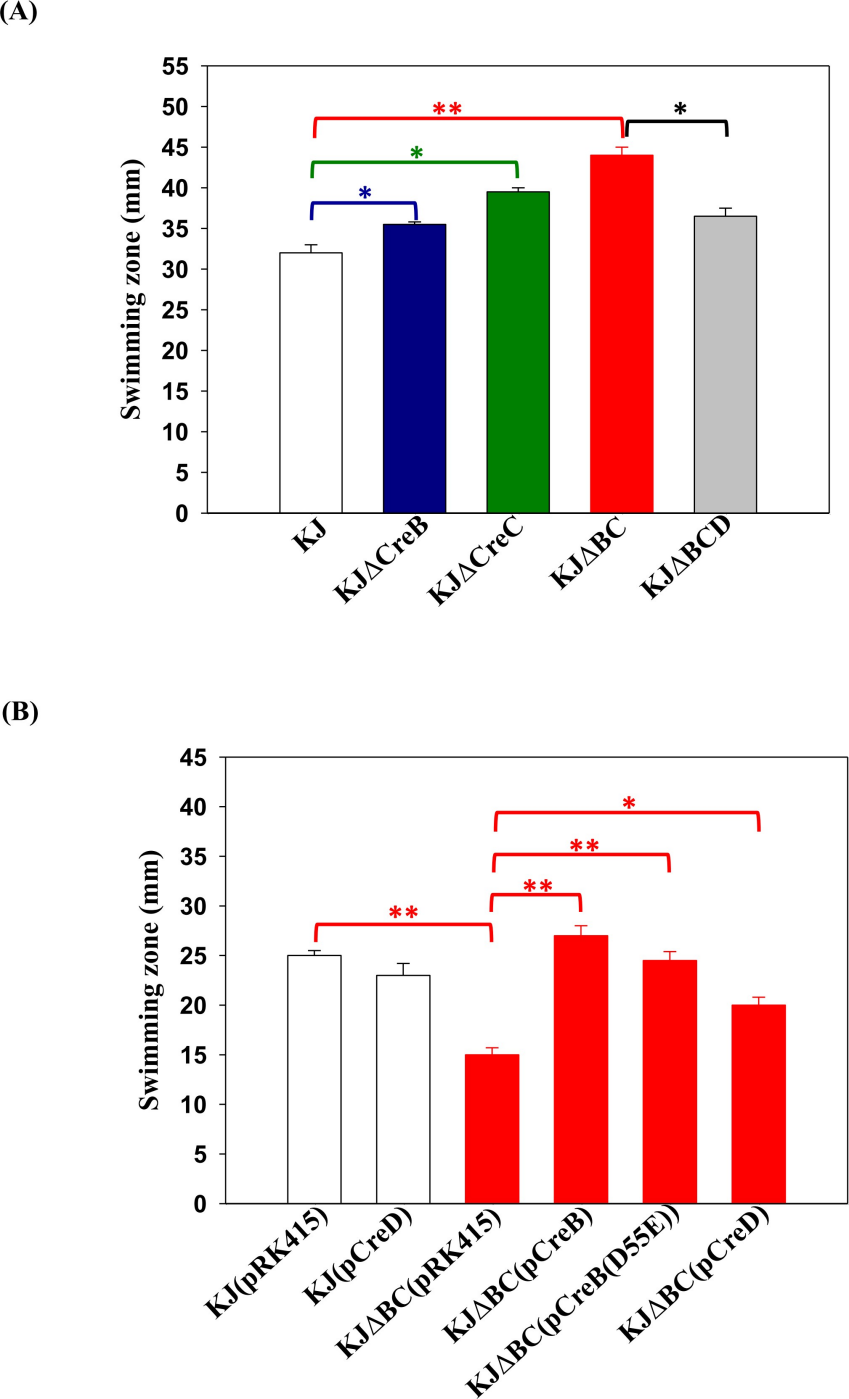
The role of CreBC TCS in the swimming motility. Two microliters of bacterial cell suspension was inoculated onto the swimming agar (1% tryptone, 0.5% NaCl, and 0.15% agar). Results were expressed as diameters (millimetres) of swimming zones after 48 h of incubation at 37°C. Data represent the means from 3 independent experiments. Error bars represent the standard deviations for three triplicate samples. *, *p* < 0.01; **, *p* < 0.001. (A) The swimming motility of *creB*, *creC*, *creBC*, and *creBCD* mutants. (B) The swimming motility of *creBC* mutant and its derived complementation strains. Tetracycline of 30 μg/ml was added for the plasmid maintenance.

The swimming motilities of KJΔCreB, KJΔCreC, KJΔBC, and wild-type KJ were examined by assessing their migration through semi-solid agar (0.15% agar). Compared to wild-type KJ, KJΔCreB and KJΔCreC displayed enhanced swimming motility, and this phenotype was exacerbated through simultaneously deletion of CreB and CreC, namely KJΔBC (Fig 3A). Next, the complementation assay was performed to further confirm the involvement of the CreBC TCS in swimming motility. The empty vector pRK415 was introduced into KJ and KJΔBC as a control. To our surprise, the swimming zone of KJΔBC(pRK415) was smaller than that of KJ(pRK415) (Fig 3B), opposite to the results observed for KJΔBC and KJ strains (Fig 3A). The phenotypic deviation mediated by plasmid introduction indicates that the maintenance of plasmid pRK415 in KJ strain may alter the expressions of *creBC*-regulated genes, which are responsible for the swimming phenotype. This is reminiscent of *creD*, whose expression is negatively regulated by CreBC TCS and positively regulated by the bacterial culture density [14]. Factors that decrease the bacterial culture density, such as plasmid carrying and tetracycline addition for plasmid maintenance, may attenuate the *creD* expression [14]. A speculated model was thus proposed herein that CreD upregulation in KJΔBC mutant may contribute to the enhanced swimming motility in KJΔBC, as observed for KJ and KJΔBC (Fig 3A). However, CreD upregulation in KJΔBC was counteracted by the introduction of plasmid pRK415 and the addition of tetracycline [14], which may account for the swimming motility observations in KJ(pRK415) and KJΔBC(pRK415) (Fig 3B). To assess this, we first determined the *creD* transcript levels in KJ and KJΔBC, as well as in KJ(pRK415) and KJΔBC(pRK415). Consistent with previous results [14], the *creD* transcript showed a 2.67±1.02-fold increase in KJΔBC compared to that in wild-type KJ. However, the *creD* transcript was decreased in plasmid-carriage strains (KJ(pRK415) and KJΔBC(pRK415)) (S1 Fig.), signifying that plasmid introduction and tetracycline addition attenuate the promoter activity of *P*_*creD*._, consistent with our previous finding [14] Next, three strategies were adopted to link CreD to the swimming phenotype: (i) a Δ*creD* allele was introduced into KJΔBC, yielding KJΔBCD, (ii) KJΔBC was complemented with *creB-* and *creB*(D55E)-containing plasmids, yielding KJΔBC(pCreB) and KJΔBC(pCreB(D55E)) respectively, and (iii) a *creD*-containing plasmid was introduced into KJΔBC and wild-type KJ, generating KJΔBC(pCreD) and KJ(pCreD) respectively. The swimming zone of KJΔBCD was smaller than that of KJΔBC, but not as small as that of wild-type KJ (Fig 3A), indicating that *creD* upregulation in the Δ*creBC* background partially contributed to enhanced swimming. In the plasmid-harbouring counterpart, swimming motility alterations caused by *creBC* inactivation were reverted to wild-type levels when either CreB or CreB(D55E) was complemented (Fig 3B). Furthermore, the swimming zone of KJΔBC(pCreD) was larger than that of KJΔBC(pRK415), but not as large as that of KJ(pRK415) (Fig 3B). However, overexpression of CreD in the wild-type KJ had only a minor effect on swimming motility (Fig 3B, KJ(pRK415) vs. KJ(pCreD)). Therefore, CreD overexpression is not the sole parameter contributing to enhanced swimming of KJΔBC. Some factors in Δ*creBC* background, but not in the wild-type background, are involved swimming enhancement. Taken together, these data support that (i) swimming motility in *S*. *maltophilia* is negatively regulated by CreBC TCS; (ii) *ΔcreBC*-mediated CreD upregulation partially contributes to increased swimming motility in KJΔBC; and (iii) in addition to *creD*, other genes regulated by CreBC TCS contribute to enhanced motility in KJΔBC.

### Inactivation of *creBC* upregulates the expression of flagella-associated genes encoding the basal body and motor-associated proteins

The flagellum is a critical organelle for bacterial swimming motility. Flagella morphologies in KJ and KJΔBC cells were further assessed to elucidate the possible link between flagella morphology and motility. Most KJΔBC cells maintained similar flagella numbers and morphologies compared to those of KJ cells (Fig 1B), although we observed a few instances in which KJΔBC cells had more flagella. The minority of hyper-flagella KJΔBC cells was likely not the main cause of enhanced swimming in KJΔBC cells.

To further clarify the underlying mechanism of Δ*creBC*-mediated swimming enhancement, transcriptome sequencing (RNA-seq) was performed to examine gene expression in KJ and KJΔBC cells. For analysis, we defined differentially expressed genes as those with an absolute fold change greater than 3. The transcriptome data revealed that 723 genes were differentially regulated between KJ and KJΔBC (S1 Table). Of these, 673 (93%) and 50 (7%) were upregulated and downregulated, respectively, in response to *creBC* inactivation, indicating that the CreBC TCS mainly acts as gene repressor in wild-type KJ.

**Fig 4:**
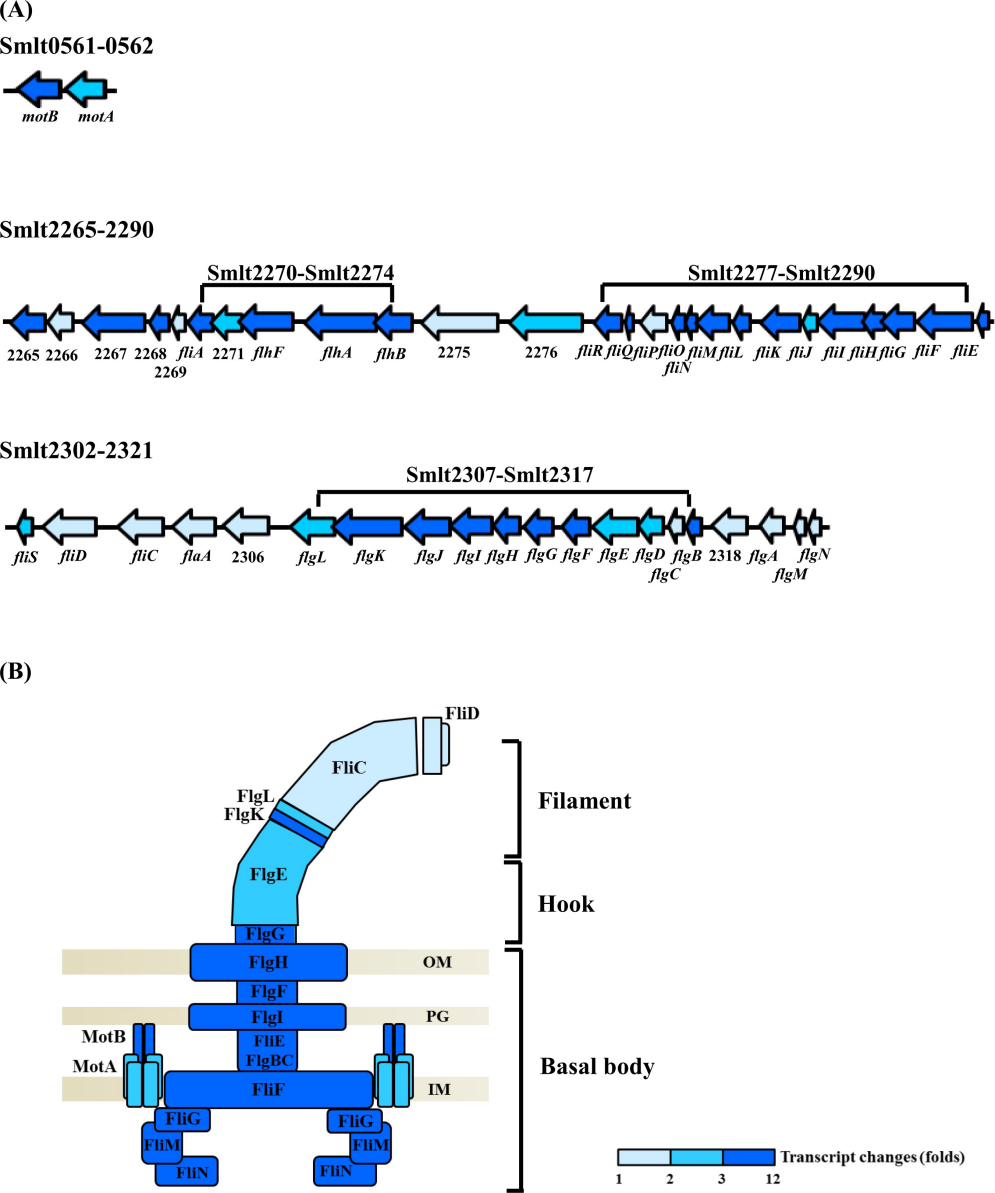
The transcript changes (folds) of flagella-related genes between KJ and KJΔBC cells by transcriptome analysis. Total mRNA was extracted from KJ and KJΔBC logarithmic-phase cultures. The ribosomal RNA (rRNA) depletion, adapter-ligated cDNA library construction and enrichment, and cDNA sequencing were performed as described in Materials and Methods. Transcript changes (folds) of a gene is expressed as the transcript in KJΔBC relative to the transcript in wild-type KJ (KJΔBC/KJ). Navy blue color indicates the transcript change of the gene is greater than or equal to 3. Blue color indicates the transcript change of the gene is less than 3 and greater than 2. Light blue color indicates the transcript change of the gene is less than 2 and greater than 1. (A) The genomic organizations of the flagella-related genes. Based on the transcriptome assay, the flagella-related genes, upregulated in case of *creBC* inactivation, are located in three clusters, Smlt0561-0562, Smlt2265-2290, and Smlt2302-2321. The orientation of gene is indicated by the arrow. (B) Schematic diagram of bacterial flagellum. The flagellum consists of the basal body, the hook, and the filament. The composition proteins are labelled. OM, outer membrane; PG, peptidoglycan layer; IM, inner membrane.

From the transcriptome results, we noticed that a cohort of putative flagella-related genes was significantly upregulated with *creBC* inactivation. These genes are located in three clusters of the *S*. *maltophilia* genome, Smlt0561–Smlt0562, Smlt2265–Smlt2290, and Smlt2302–2321 (Fig 4A). After further classifying upregulated genes, we noticed that proteins encoded by the Smlt0561–0562, Smlt2277–2290, and Smlt2307–2317 clusters comprised the stator, C-ring/MS-ring/motor switch, and P-ring/L-ring/rod/hook, respectively (Fig 4B). Of note, the expression of Smlt2270 (*fliA*), encoding the putative RNA polymerase sigma factor FliA for flagella regulon, and Smlt2272 (*flhF*), encoding the putative flagella biosynthesis regulator FlhF, were elevated by 5.33-fold and 9.42-fold, respectively, in KJΔBC cells compared to that in KJ cells. In addition, whereas *creBC* was inactivated, the upregulation of genes encoding rotor proteins (FliF, FliG, FliM, and FliN) and stator proteins (MotA and MotB) ranged from 2.64- to 7.37-fold (Table 1). Nevertheless, it was noticed that the expression of *fliC* and *fliD*, which encode filament and filament cap proteins, respectively, was not significantly altered when CreBC was inactivated (Table 1 and Fig 4).

**Table 1:** Flagella-associated genes differently expressed in *S*. *maltophilia* KJ and KJΔBC cells.

**Locus**	**Normalized expression**		**Fold change RNAseq**	**Encoded protein**
	KJ	KJΔBC		
Smlt0561	12.47	49.33	3.95	flagellar motor protein MotB
Smlt0562	40.50	108.31	2.64	flagellar motor protein MotA
Smlt2265	10.09	39.25	3.88	flagellar motor protein MotD
Smlt2266	24.16	45.68	1.89	flagellar motor protein
Smlt2267	9.92	46.19	4.62	two component sensor kinase
Smlt2268	16.94	64.45	3.80	chemotaxis protein
Smlt2269	18.76	31.80	1.69	two component response regulator
Smlt2270	8.14	43.39	5.33	RNA polymerase sigma factor, FliA
Smlt2271	17.64	47.89	2.71	ParA family ATPase flagella number regulator
Smlt2272	7.78	73.37	9.42	flagellar biosynthesis regulator, FlhF
Smlt2273	4.93	32.43	6.57	flagellar biosynthesis protein FlhA
Smlt2274	6.63	28.54	4.30	flagellar biosynthesis protein FlhB
Smlt2275	40.32	67.55	1.67	esterase/peptidase
Smlt2276	11.89	27.22	2.28	transmembrane GGDEF EAL domain signaling protein
Smlt2277	3.74	22.42	5.99	flagellar biosynthetic protein FliR
Smlt2278	1.95	21.86	11.20	flagellar biosynthetic protein FliQ
Smlt2279	11.81	19.14	1.61	flagellar biosynthesis protein FliP
Smlt2280	4.70	45.87	9.75	flagellar protein FliO
Smlt2281	3.52	14.98	4.24	flagellar rotor switch protein FliN
Smlt2282	6.15	21.93	3.56	flagellar rotor switch protein FliM
Smlt2283	4.84	16.82	3.46	flagellar basal body-associated protein FliL
Smlt2284	11.31	59.35	5.24	flagellar hook-length control protein FliK
Smlt2285	11.25	27.44	2.43	flagellar FliJ protein
Smlt2286	6.55	48.97	7.47	flagellum-specific ATP synthase FliI
Smlt2287	5.53	27.03	4.88	flagellar assembly protein FliH
Smlt2288	6.83	24.42	3.57	flagellar rotor switch protein FliG
Smlt2289	5.43	29.72	5.46	flagellar MS-ring protein FliF
Smlt2290	6.47	22.29	3.44	flagellar hook-basal body complex protein FliE
Smlt2302	16.41	47.87	2.91	flagellar protein FliS
Smlt2303	23.84	39.02	1.63	flagellar hook-associated protein FliD
Smlt2304	75.80	77.76	1.02	Flagellin FliC
Smlt2305	21.09	30.04	1.42	flagellin FlaA
Smlt2306	32.67	59.85	1.83	flagellin
Smlt2307	12.47	25.68	2.05	flagellar hook-associated protein FlgL
Smlt2308	13.92	45.03	3.23	flagellar hook-associated protein FlgK
Smlt2309	8.96	44.14	4.92	flagellar rod assembly protein/muramidase FlgJ
Smlt2310	10.13	35.53	3.50	flagellar basal body P-ring protein FlgI
Smlt2311	10.83	43.08	3.97	flagellar basal body L-ring protein, FlgH
Smlt2312	11.89	36.44	3.06	flagellar basal body rod protein FlgG
Smlt2313	8.07	24.53	3.03	flagellar basal body rod protein FlgF
Smlt2314	15.49	39.00	2.51	flagellar hook protein FlgE
Smlt2315	9.24	22.33	2.41	flagellar basal body rod modification protein FlgD
Smlt2316	12.90	16.16	1.25	flagellar basal body rod protein FlgC
Smlt2317	7.98	24.11	3.02	flagellar basal body rod protein FlgB
Smlt2318	15.18	24.79	1.63	two-component response regulator chemotaxis signal
Smlt2319	34.26	55.22	1.61	flagellar basal body P-ring biosynthesis protein FlgA
Smlt2320	192.76	195.71	1.01	FlgM
Smlt2321	57.48	94.73	1.64	flagella protein FlgN

**Fig 5:**
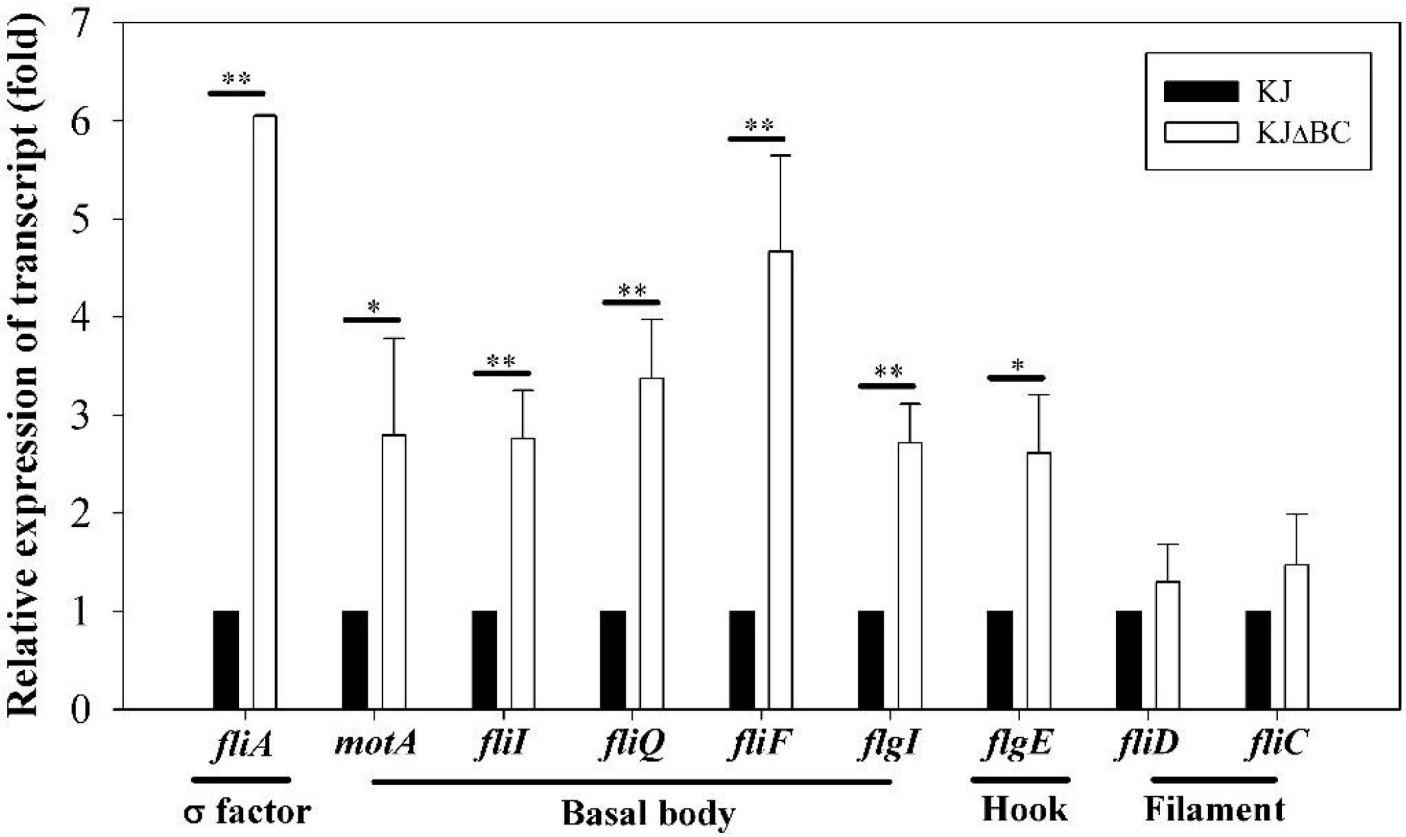
The transcript changes (folds) of selected flagella-related genes between KJ and KJΔBC cells by qRT-PCR. Total mRNA was extracted from KJ and KJΔBC logarithmic-phase cultures. cDNA was prepared by RT-PCR and used as the template for qRT-PCR. The expression of target gene transcripts in qRT-PCR were normalized to the level of expression of the 16S rRNA gene by using the ΔΔ*C*_*T*_ method. Data are the means from three independent experiments. Error bars represent the standard deviations for three triplicate samples. *, *p* < 0.05; **, *p* < 0.005.

A set of nine flagella-related genes was chosen for validation by qRT-PCR, including Smlt0562 (*motA*), Smlt2270 (*fliA*), Smlt2278 (*fliQ*), Smlt2286 (*fliI*), Smlt2289 (*fliF*), Smlt2303 (*fliD*), Smlt2304 (*fliC*), Smlt2310 (*flgI*), and Smlt2314 (*flgE*) (Fig 5). Overall, the results of qRT-PCR analysis were in good agreement with the transcriptome data (Table 1).

### CreD upregulation in KJΔBC alleviates the *ΔcreBC*-mediated membrane integrity compromise

**Fig 6:**
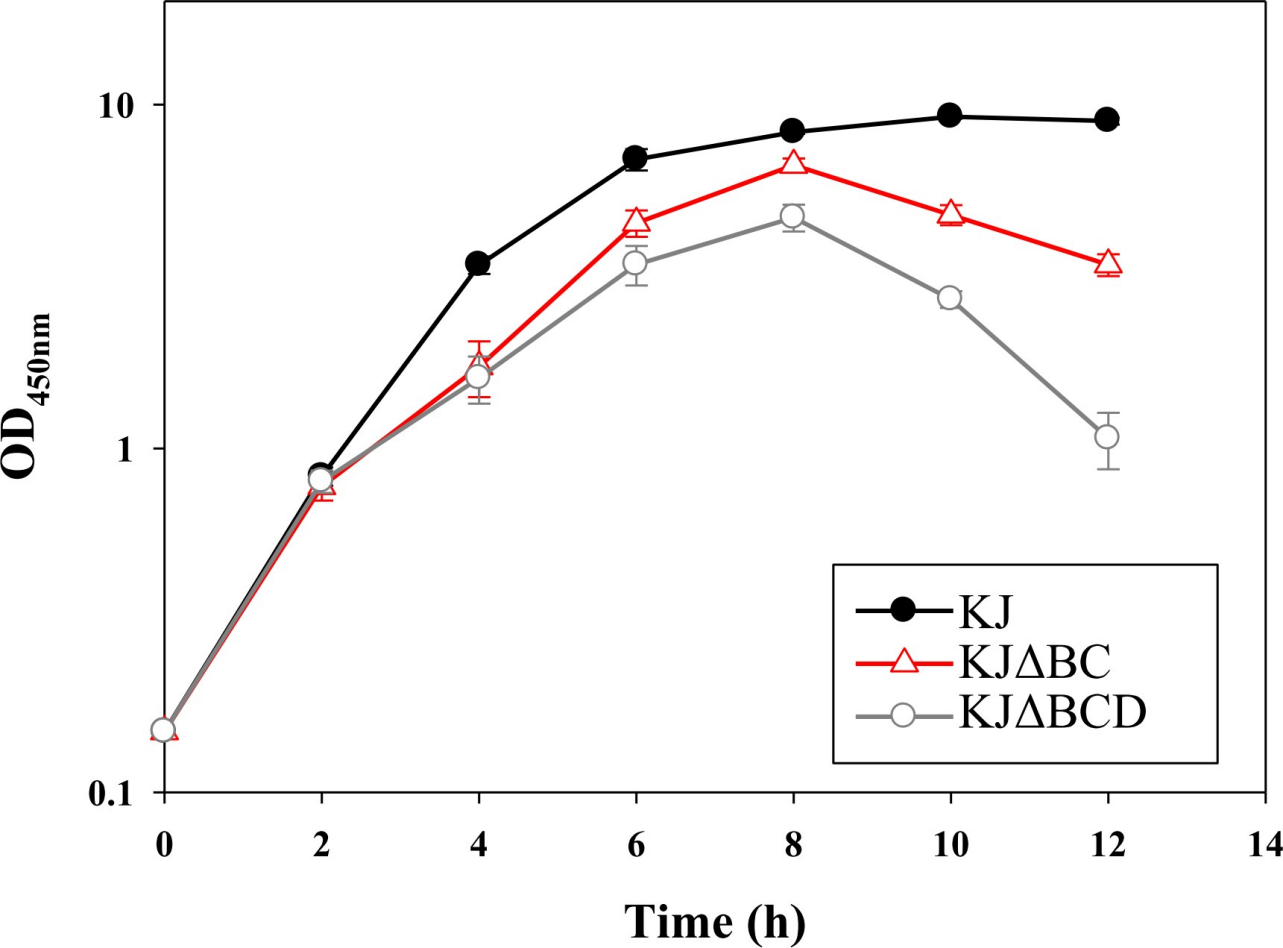
The roles of *creBC* and *creD* in membrane susceptibility to Triton X-100. The overnight-cultured bacteria were inoculated into fresh LB broth containing Triton X-100 of 200 μg/ml at the initial OD_450_ of 0.15. The bacterial growth was monitored by recording the OD_450nm_. Data are the means from three independent experiments. Error bars indicate the standard deviations for three triplicate samples.

The observation that the deletion of the *creD* allele from KJΔBC compromised swimming motility (Fig 3A) supports the involvement of CreD in the Δ*creBC*-mediated increase in swimming motility. CreD is an inner membrane protein with six transmembrane α-helix domains (http://www.cbs.dtu.dk/services/TMHMM/). In our recent study, we showed that the *creD* mutant, KJΔCreD, has cell division defects and aberrations in cell envelope integrity [14], strengthening the possibility that CreD acts in the architectural frame of the inner membrane and plays a critical role in the maintenance of membrane integrity. Intact membrane architecture is essential for the successful assembly and function of the flagellum. Therefore, we considered whether CreD upregulation in KJΔBC cells contributes to the maintenance of membrane integrity, inferring a relationship between CreD and swimming motility. Membrane susceptibility of KJ, KJΔBC, and KJΔBCD to the detergent triton X-100 was assessed. The growth of KJΔBC in the presence of triton X-100 was compromised compared to that of wild-type KJ, and this impairment was further exacerbated when the *ΔcreD* allele was introduced into the chromosome of KJΔBC (Fig 6). This observation supports the contention that CreD upregulation in KJΔBC alleviates the *ΔcreBC*-mediated membrane integrity compromise, which might benefit flagellum construction and swimming motility.

### KJΔBC cells show elevated CCCP_50_ value compared to KJ cells

The inner membrane of bacterial cells harbors a membrane potential, which is formed by the differences in the concentrations of ions on opposite sides of an inner membrane. One of the functions of the membrane potential is to provide power to operate flagellum rotation and drive swimming [19]. Given the upregulation of genes encoding rotor and stator proteins in KJΔBC cells (Figs 4 and 5), we evaluated whether upregulation of stator proteins in KJΔBC cells would provide a higher membrane potential, contributing to enhanced swimming. Carbonyl cyanide *m*-chlorophenyl hydrazone (CCCP), a chemical inhibitor of oxidative phosphorylation, causes uncoupling of the proton gradient and thus abolishes swimming motility [20]. Therefore, we determined the CCCP_50_ of KJ cells and KJΔBC cells to determine to what extent membrane potential contributes to swimming. CCCP_50_ is defined as the CCCP concentration at which 50% of bacterial swimming is inhibited. We found that inactivation of *creBC* increased the CCCP_50_ value by approximately 1.3-fold (Table 2). Next, a complementation assay was performed. As a control, we also introduced the empty pRK415 vector into KJ and KJΔBC. Surprisingly, empty vector introduction had an opposite effect on CCCP_50_ values; the CCCP_50_ value of KJΔBC(pRK415) was lower than that of KJ(pRK415) (Table 2). A similar observation for plasmid introduction-mediated phenotypic deviation was obtained in the aforementioned swimming phenotype (Fig 3B). The plasmid introduction-mediated phenotypic deviation will be discussed below. Complementation of KJΔBC with pCreB or pCreB(D55E) reverted the CCCP_50_ value to the wild-type level (Table 2), supporting that *creB*-mediated signalling affects the membrane potential. Furthermore, we found that the CCCP_50_ values of the strains assayed (Table 2) were positively correlated with swimming motility (Fig 3).

**Table 2:** The CCCP_50_ values of *S*. *maltophilia* KJ, its isogenic *creBC* mutant (KJΔBC), and the complementary strains.

Strain	CCCP_50_^a^ (μg/ml)
KJ	33.4 ± 1.0
KJΔBC	43.2 ± 0.4
KJ(pRK415)	31.4 ± 1.1
KJΔBC(pRK415)	24.4 ± 0.8
KJΔBC(pCreB)	39.8 ± 8.5
KJΔBC(pCreB(D55E))	31.9 ± 1.8

^a^ CCCP_50_ is defined as the required CCCP concentration at which the fifty percent of bacterial swimming is inhibited.

## Discussion

The CreBC/BlrAB TCS in *E*. *coli*, *Aeromonas* spp., and *P*. *aeruginosa* is considered a defense system, helping bacteria to alleviate stresses, since it is activated by certain pressures such as anaerobic environments or β-lactam challenge. However, the CreBC of *S*. *maltophilia* is constitutively active in laboratory LB-cultured conditions without foreign stresses, signifying its possible role in the maintenance of bacterial physiology not limited to stress defense. Our results in this study provide evidence that the CreBC TCS of *S*. *maltophilia* negatively modulates swimming motility.

Swimming motility is an important mechanism for bacterial survival, allowing bacteria to approach nutrient sources, invade host cells, and escape from attack [21]. Nevertheless, swimming motility is an energy-consuming process, and inefficiencies result in energy waste, which curtails utilizable energy for bacterial growth [22]. Therefore, exquisitely modulating swimming motility is critical for bacterial survival in different environmental niches. It has been reported that TCSs generally act as positive regulators of swimming motility in response to environmental stimuli. Examples of this include the BceSR TCS of *Burkholderia cenocepacia*, the RpfCG TCS of *Xanthomonas albilineans*, and the QseBC TCS of *Aeromonas hydrophila* [23–25]. In a recent study, Zheng et al. successfully constructed 51 histidine kinase (HK) mutants of *S*. *maltophilia* and swimming motility was assessed in these mutants. Of 51 HK mutants, six had deficiency in swimming motility (not caused by growth defects) and no mutations were found to enhance swimming motility [7]. Unfortunately, a *creC* mutant could not be successfully constructed and therefore was not included in their assays. However, their findings provided an indication that at least six constitutively active TCS systems positively regulate swimming motility in *S*. *maltophilia* in the laboratory-cultured conditions [7]. In this study, we successfully constructed the *creBC* mutant and verified the role of the CreBC TCS in the negative regulation of swimming motility.

Examples of TCS systems acting as negative modulators of swimming motility have seldom been reported, with the exception of the GacS-GacA systems of *P*. *fluorescens* F113 and *P*. *chlororaphis* O6 [26–27]. The underlying mechanisms of GacSA TCS regulation of swimming motility are attributed to increasing flagella elongation [26] or flagella numbers [27]. In this study, we provide another example of a TCS (specifically CreBC) negatively regulating swimming motility in *S*. *maltophilia*, as a mutation in the *creBC* genes resulted in increased motility (Fig 3A). Distinct from GacS-GacA in *Pseudomonas* spp., CreBC inactivation-mediated swimming motility in *S*. *maltophilia* might result from increased motor output of the flagellum, rather than alterations in flagella numbers or morphology. This inference was supported by the observations that the motor-associated genes (*motA*, *motB*, *fliG*, *fliM*, *fliN*, and *fliQ*) were highly upregulated and the helical filament-associated genes (*fliC* and *fliD*) were normally expressed in KJΔBC (Table 1, Figs 4 and 5). Furthermore, the membrane potential of KJΔBC cells was higher than that of wild-type KJ cells (Table 2).

Interestingly, we found that some phenotypes of the KJΔBC mutant showed dramatically changes when an empty vector (pRK415) was introduced, including the swimming motility (Fig 3B) and CCCP_50_ value (Table 2). There are two possible explanations for this as follows: (i) plasmid introduction may affect the expression of some genes in the CreBC regulon, and these genes affect the phenotypes assayed. CreD, a member of the CreBC regulon, is such an example. (ii) Plasmid carriage is an energy-consuming process, which may redistribute the energy utilization in bacteria and thus alter some energy-dependent phenotypes. This may also explain why some complementation assays performed by ectopic expression of the mutated genes in this study could not fully restore the phenotypes of the mutant to the wild-type level.

We have previously indicated that *creD* expression is upregulated in response to CreBC inactivation [14]. In this study, we further demonstrated that CreD upregulation makes a significant contribution to cell membrane integrity in KJΔBC (Fig 6). The relevance of CreD upregulation and cell membrane integrity for enhanced swimming motility are highlighted by the fact that enhanced swimming motility and cell membrane integrity in KJΔBC are compromised by *creD* inactivation (Figs 3A and 6). According to these observations, we propose a model for the negative regulatory role of the CreBC TCS in swimming motility in *S*. *maltophilia*. The CreBC TCS is constitutively active, signifying its importance in bacterial physiology. Activated CreB maintains the expression of flagella-associated genes at an adequate level, especially the membrane proton flow related genes (*motA* and *motB*), preventing inefficient swimming and thus refining bacterial energy utilization in *S*. *maltophilia*. In the absence of a functional CreBC TCS, some physiological functions of *S*. *maltophilia* are compromised, such as secreted protease activity (Fig 2A) and oxidative stress tolerance (our unpublished data), threatening bacterial survival during stress challenge. Nevertheless, *ΔcreBC*-mediated swimming motility enhancement could provide a survival benefit for KJΔBC by effectively escaping from stresses. The underlying mechanisms for enhanced swimming motility in KJΔBC may involve the upregulation of *creD* and increased membrane potentials. Although the exact reason for the membrane potential elevation of KJΔBC cells remains unclear, upregulation of flagella-associated genes encoding basal body- and motor-associated proteins may be involved. Increased CreD enforces the membrane integrity, which is critical for flagellum assembly and motility.

## Materials and methods

### Bacterial strains and culture conditions

A complete list of strains, plasmids, and primers used in this study is shown in S2 Table. *S*. *maltophilia* KJ acts as the parental wild type strain [28]. Cells were grown at 37°C in Luria-Bertani (LB) broth.

### Construction of deletion mutants KJΔCreB and KJΔCreC

The KJΔCreB and KJΔCreC in-frame deletion mutants were constructed by double-crossover homologous recombination between the wild-type KJ chromosome and plasmids pΔCreB and pΔCreC, respectively. The pΔCreB was prepared as follows: the intact *creB* gene was amplified from the wild-type KJ chromosome using the primers CreB-F and CreB-R (S2 Table) and cloned into pEX18Tc, yielding pEXCreB. Plasmid pEXCreB was digested by PstI and then self-ligated to generate pΔCreB, in which the internal 402-bp PstI-PstI fragment of *creB* was deleted. The pΔCreC was prepared as follows: two DNA fragments targeting the 5’ terminus and the 3’ terminus of the *creC* genes were obtained by PCR using the primer sets CreCN-F/CreCN-R and CreCC-F/CreCC-R (S2 Table). The PCR amplicons were digested and subsequently cloned into pEX18Tc. Plasmid mobilization, transconjugants selection, and mutant confirmation were performed as described previously [29]. The 44 to 177 amino acids of CreB and the 4 to 359 amino acids of CreC were thus deleted in the in-frame deletion mutants KJΔCreB and KJΔCreC respectively.

### Plasmids construction

The plasmids pCreB and pCreC were constructed by cloning *creB* and *creC* amplified with primers CreB-F/CreB-R and CreCN-F/CreCC-R into the pRK415, respectively. The *creB(D55E)* allele was generated by site-directed mutagenesis using primer extension PCR as described previously [14] and cloned into pRK415 at HindIII/XbaI sites to generate pCreB(D55E). All constructs were verified by sequencing. All primers are listed in S2 Table in the supplemental materials.

### Scanning electron microscope (SEM)

The bacterial cells for SEM observation were prepared as described previously^14^. Briefly, the bacterial cells (OD_450nm_ of 1.0) were harvested by centrifugation and washed three times with PBS (pH7.4). The bacteria were pre-fixed with 2.5% glutaraldehyde in 0.1 M phosphate buffer (pH 7.4), washed, post-fixed with 1% osmium tetraoxide (OsO_4_), and then dehydrated by ethanol. The high-resolution FEI Inspect S scanning electron microscope was used for the observation of bacterial cells.

### Secreted protease activity assay

The secreted protease activity of bacteria was assayed using LB agar containing 1% skim milk. For the convenience of bacterial suspension loading, the skim milk agar was prepared with a 6-mm-diameter hole in the center. The overnight cultured bacteria were adjusted to an OD_450_ of 1.0 and 40 μl of the bacterial suspension was dripped onto the hole of the skim milk agar plates. After incubation at 37°C for 72 h, the secreted protease activity of bacteria was assessed by measuring the protease hydrolyzing zones around the bacteria.

### Swimming assay

The bacterial strains tested were grown to an OD_450_ of 1.0, and 2 μl of bacterial suspension was inoculated at the swimming agar surface (1% tryptone, 0.5% NaCl, and 0.15% agar) [30]. The plates were incubated at 37°C for 48 h. Results are expressed as diameters (millimeters) of swimming zones. For the determination of CCCP_50_, swimming assay was performed using the swimming agar containing the CCCP of 0,10, 20, 30, 40, 50, and 60 μg/ml, respectively. CCCP_50_ is defined as the CCCP concentration at which 50% of bacterial swimming is inhibited.

### Flagella staining

The bacterial strains tested were grown to an OD_450_ of 1.0. And the bacterial suspension was diluted twice with PBS (pH 7.4). The bacterial suspension was negatively stained with 1% phosphotungstic acid (pH 7.4) on Formvar-coated copper grids [29]. The presence of flagella was observed by transmission electron microscope (TEM) (Hitachi H-7650 microscope).

### Transcriptome sample preparation and sequencing

Total RNA isolation, ribosomal RNA (rRNA) depletion, adapter-ligated cDNA library construction and enrichment, and cDNA sequencing were performed as described previously [6]. After trimming of low quality of bases (< Q30), the first 12 bases and adapters, the trimmed Reads were mapped to the *Stenotrophomonas maltophilia* K279a genome (GenBank acc. no. NC_010943.1) and run RNA-seq analysis by CLC Genomics Workbench v 6.0 (CLC Bio). RNA-seq data representing the alignment of sequences (short reads) to coding sequences (CDS) were quantified as reads per kilobase CDS length per million reads analyzed (RPKM). The sequence dataset was deposited in NCBI Sequence Read Archive (SRA) database under STUDY accession number SRP100809.

### Quantitative real-time PCR (qRT-PCR)

Total cellular DNA-free RNA extraction, cDNA preparation, and the transcripts of the flagella-related genes determination were carried out as described previously [29]. A complete list of primers used for qRT-PCR is shown in S2 Table. The 16S rRNA gene was used as the normalizing gene. The relative expression of mRNA from each gene of interest was determined by the comparative cycle threshold (*C*_*T*_) method [31].

### Triton X-100 susceptibility test

The envelope integrity of bacteria was assessed by evaluating the bacterial capability to protect the membranes against Triton X-100. Overnight cultures of the bacteria strains were diluted to an OD_450_ of 0.15 with LB broth containing 200 μg/ml Triton X-100. The OD_450nm_ was read at an interval of 2 h.

## Supporting information

S1 Fig Expression of *creD* genes for strain KJ and its derived strains, determined by qRT-PCR. (JPG)

S1 Table Genes differently expressed in *S*. *maltophilia* KJ and KJΔBC cells. (DOCX)

S2 Table Bacterial strains, plasmids and primers used in this study. (DOCX)

## References

[1] Brooke JS. *Stenotrophomonas maltophilia*: an emerging global opportunistic pathogen. Clin Microbiol Rev. 2012;25: 2–41. doi: 10.1128/CMR.00019-11 22232370 10.1128/CMR.00019-11 PMC3255966

[2] Krell T, Lacal J, Busch A, Silva-Jimenez H, Guazzaroni ME, Ramos JL. Bacterial sensor kinases: diversity in the recognition of environmental signals. Annu Rev Microbiol. 2010;64: 539–559. doi: 10.1146/annurev.micro.112408.134054 20825354 10.1146/annurev.micro.112408.134054

[3] Gao R, Stock AM. Biological insights from structures of two-component protein. Annu Rev Microbiol. 2009;63: 133–154. doi: 10.1146/annurev.micro.091208.073214 19575571 10.1146/annurev.micro.091208.073214 PMC3645274

[4] Crossman LC, Gould VC, Dow JM, Vernikos GS, Okazaki AK, Sebaihia M, et al The complete genome, comparative and functional analysis of *Stenotrophomonas maltophilia* reveals an organism heavily shielded by drug resistance determinants. Genome Biol. 2008;17: R74. 10.1186/gb-2008-9-4-r74 PMC2643945 18419807

[5] Li XZ., Zhang L, Poole K. SmeC, an outer membrane multidrug efflux protein of *Stenotrophomonas maltophilia*. Antimicrob Agents Chemother. 2002;46: 333–343. doi: 10.1128/AAC.46.2.333-343.2002 11796339 10.1128/AAC.46.2.333-343.2002 PMC127032

[6] Wu CJ, Huang YW, Lin YT, Ning HC, Yang TC. Inactivation of SmeSyRy two-component regulatory system inversely regulates the expression of SmeYZ and SmeDEF efflux pumps in *Stenotrophomonas maltophilia*. PLoS One. 2016;11: e0160943 doi: 10.1371/journal.pone.0160943 27513575 10.1371/journal.pone.0160943 PMC4981351

[7] Zheng L, Wang FF, Ren BZ, Liu W, Liu Z, Qian W. Systematic mutational analysis of histidine kinase genes in the nosocomial pathogen *Stenotrophomonas maltophilia* identifies BfmAK system control of biofilm development. Appl Environ Microbiol. 2016;82: 2444–2456. doi: 10.1128/AEM.03951-15 26873318 10.1128/AEM.03951-15 PMC4959488

[8] Avison MB, Horton RE, Walsh TR, Bennett PM. *Escherichia coli* CreBC is a global regulator of gene expression that responds to growth in minimal media. J Biol Chem. 2001;276: 26955–26961. doi: 10.1074/jbc.M011186200 11350954 10.1074/jbc.M011186200

[9] Moya B, Dotsch A, Juan C, Blazquez J, Zamorano L, Haussler S, et al β-lactam resistance response triggered by inactivation of a nonessential penicillin-binding protein. PLoS Pathog. 2009;5: e1000353 doi: 10.1371/journal.ppat.1000353 19325877 10.1371/journal.ppat.1000353 PMC2654508

[10] Tayler AE, Ayala JA, Niumsup P, Westphal K, Baker JA, Zhang L, et al Induction of β-lactamase production in *Aeromonas hydrophilia* is responsive to β-lactam-mediated changes in peptidoglycan composition. Microbiology 2010;156: 2327–2335. doi: 10.1099/mic.0.035220-0 20430811 10.1099/mic.0.035220-0

[11] Alksne LE, Rasmussen BA. Expression of the AsbA1, OXA-12, and AsbM1 β-lactamases in *Aeromonas jandaei* AER 14 is coordinated by a two-component regulon. J Bacteriol. 1996;179: 2006–2013. 10.1128/jb.179.6.2006-2013.1997 PMC178926 9068648

[12] Niumsup PA, Simm AM, Nurmahomed K, Walsh TR, Bennett PM, Avison MB. Genetic linkage of the penicillinase gene, *amp*, and *blrAB*, encoding the regulator of β-lactamase expression in *Aeromonas* spp. J Antimicrobl Chemother. 2003;51: 1351–1358. 10.1093/jac/dkg247 12746371

[13] Zamorano L, Moya B, Juan C, Mulet X, Blazquez J, Oliver A. The *Pseudomonas aeruginosa* CreBC two-component system plays a major role in the response to β-lactam, fitness, biofilm growth, and global regulation. Antimicrob Agents Chemother. 2014;58: 5084–5095. doi: 10.1128/AAC.02556-14 24936599 10.1128/AAC.02556-14 PMC4135852

[14] Huang HH, Lin YT, Chen WC, Huang YW, Chen SJ, Yang TC. Expression and functions of CreD, an inner membrane protein in *Stenotrophomonas maltophilia*. PLoS One. 2015;10: e0145009 doi: 10.1371/journal.pone.0145009 26698119 10.1371/journal.pone.0145009 PMC4689548

[15] Chaban B, Hughes HV, Beebv M. The flagellum in bacterial pathogens: from motility and a whole lot more. Semin Cell Dev Biol. 2015;46: 91–103. doi: 10.1016/j.semcdb.2015.10.032 26541483 10.1016/j.semcdb.2015.10.032

[16] Morimoto YV, Minamino T. Structure and function of the bi-directional bacterial flagellar motor. Biomolecules 2014;4: 217–234. doi: 10.3390/biom4010217 24970213 10.3390/biom4010217 PMC4030992

[17] Toutain CM, Zegans ME, O’Toole GA. Evidence for two flagellar stators and their role in the motility of *Pseudomonas aeruginosa*. J Bacteriol. 2005;187: 771–777. doi: 10.1128/JB.187.2.771-777.2005 15629949 10.1128/JB.187.2.771-777.2005 PMC543560

[18] Huang YW, Wu CJ, Hu RM, Lin YT, Yang TC. Interplay among membrane-bound lytic transglycosylase D1, the CreBC two-component regulatory system, the AmpNG-AmpDI-NagZ-AmpR regulatory circuit, and L1/L2 β-lactamase expression in *Stenotrophomonas maltophilia*. Antimicrob Agents Chemother. 2015;59: 6866–6872. doi: 10.1128/AAC.05179-14 26282431 10.1128/AAC.05179-14 PMC4604389

[19] Terashima H, Kojima S, Homma M. Flagellar motility in bacteria structure and function of flagellar motor. Int Rev Cell Mol Biol. 2008;270: 39–85. doi: 10.1016/S1937-6448(08)01402-0 19081534 10.1016/S1937-6448(08)01402-0

[20] Kanda E, Tatsuta T, Suzuki T, Taguchi F, Naito K, Inagaki Y, et al Two flagellar stators and their roles in motility and virulence in *Pseudomonas syringae* pv. tabaci 6605. Mol Genet Genomics. 2011;285: 163–174. doi: 10.1007/s00438-010-0594-8 21165649 10.1007/s00438-010-0594-8

[21] Ottemann KM, Miller JF. Roles for motility in bacterial-host interactions. Mol Microbiol. 1997;24: 1109–1117. 9218761 10.1046/j.1365-2958.1997.4281787.x

[22] Berg HC. The rotary motor of bacterial flagella. Biochemistry 2003;72:19–54. 10.1146/annurev.biochem.72.121801.161737 12500982

[23] Khajanchi BK, Kozlova EV, Sha J, Popov VL, Chopra AK. The two-component QseBC signaling system regulates in vitro and in vivo virulence of *Aeromonas hydrophilia*. Microbiology 2012;158: 259–271. doi: 10.1099/mic.0.051805-0 21998161 10.1099/mic.0.051805-0 PMC3352359

[24] Rott P, Fleites LA, Mensi I, Sheppard L, Daugrois JH, Dow JM, et al The RpfCG two-component system negatively regulates the colonization of sugar can stalks by *Xanthomonas albilineans*. Microbiology 2013;159: 1149–1159. doi: 10.1099/mic.0.065748-0 23538716 10.1099/mic.0.065748-0

[25] Merry CR, Perkins M, Mu L, Peterson BK, Knackstedt RW, Weingart CL. Characterization of a novel two-component system in *Burkholderia cenocepacia*. Curr Microbiol. 2015;70: 556–561. doi: 10.1007/s00284-014-0744-z 25519693 10.1007/s00284-014-0744-z

[26] Martinez-Granero F, Navazo A, Barahona E, Redondo-Nieto M, Rivilla R, Martin M. The Gac-Rsm and SadB signal transduction pathways converge on AlgU to downregulate motility in *Pseudomonas fluorescens*. PLoS One. 2012;7: e31765 doi: 10.1371/journal.pone.0031765 22363726 10.1371/journal.pone.0031765 PMC3282751

[27] Kim JS, Kim YH, Anderson AJ, Kim YC. The sensor kinase GacS negatively regulates flagellar formation and motility in a biocontrol bacterium, *Pseudomonas chloroaphis* O6. Plant Pathol J. 2014;30: 215–219. doi: 10.5423/PPJ.NT.11.2013.0109 25289006 10.5423/PPJ.NT.11.2013.0109 PMC4174843

[28] Hu RM, Huang KJ., Wu LT, Hsiao YJ, Yang TC. Induction of L1 and L2 β-lactamases of *Stenotrophomonas maltophilia*. Antimicrob Agents Chemother. 2008;52: 1198–1200. doi: 10.1128/AAC.00682-07 18086856 10.1128/AAC.00682-07 PMC2258547

[29] Yang TC, Huang YW, Hu RM, Huang SC, Lin YT. AmpD1 is involved in expression of the chromosomal L1 and L2 β-lactamases of *Stenotrophomonas maltophilia*. Antimicrob Agents Chemother. 2009;53: 2902–2907. doi: 10.1128/AAC.01513-08 19414581 10.1128/AAC.01513-08 PMC2704650

[30] Murray TS, Kazmiercrak BI. FlhF is required for swimming and swarming in *Pseudomonas aeruginosa*. J Bacteriol. 2006;188: 6995–7004. doi: 10.1128/JB.00790-06 16980502 10.1128/JB.00790-06 PMC1595508

[31] Livak KJ, Schmittgen TD. Analysis of relative gene expression data using real-time quantitative PCR and the 2 (delta deltaC(T)) method. Methods. 2001;25: 402–408. doi: 10.1006/meth.2001.1262 11846609 10.1006/meth.2001.1262

