# Supplementary figures and images for: Relationship of the CreBC two-component regulatory system and inner membrane protein CreD with swimming motility in Stenotrophomonas maltophilia
Source: PLoS One. 2017 Apr 24;12(4):e0174704. doi: 10.1371/journal.pone.0174704 (PMC5402928; doi:10.1371/journal.pone.0174704)

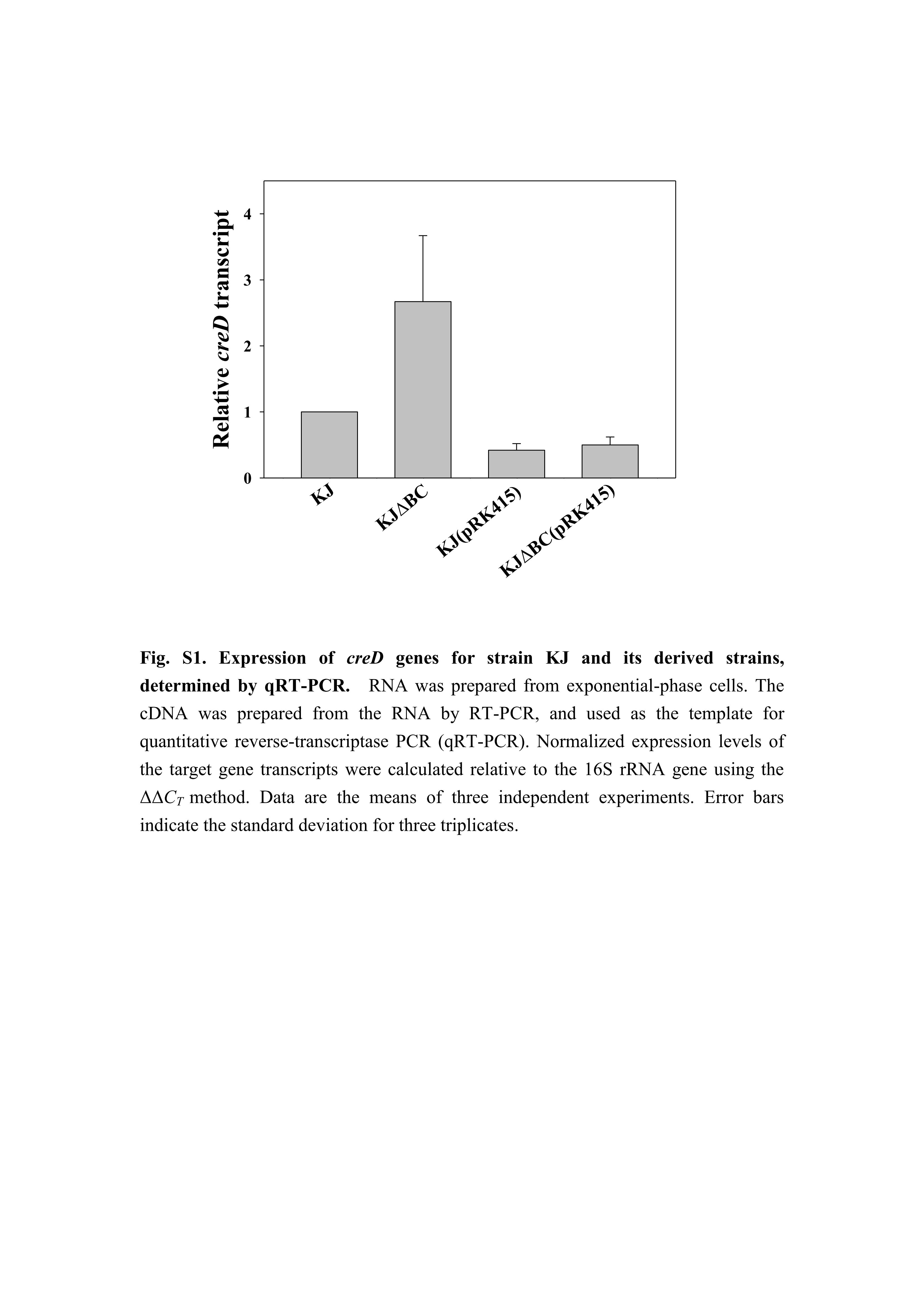

Supplement: S1 Fig — (JPG) [file pone.0174704.s001.jpg]
