# Supplementary material for: Relationship of the CreBC two-component regulatory system and inner membrane protein CreD with swimming motility in Stenotrophomonas maltophilia
Source: PLoS One. 2017 Apr 24;12(4):e0174704. doi: 10.1371/journal.pone.0174704 (PMC5402928; doi:10.1371/journal.pone.0174704)
Supplement: S1 Table — (DOCX) [file pone.0174704.s002.docx]

**S1 Table. Genes differently expressed in *S. maltophilia* KJ and KJ**Δ**BC cells**

| **Locus** | | **Normalized expression** | | | | **Encoded protein** |
| --- | --- | --- | --- | --- | --- | --- |
|  |  | **KJ** | | **KJ**Δ**BC** | |  |
| **Genes significantly upregulated in the KJ**Δ**BC cells** | | | | | | |
| Smlt0036 | | 0.336 | | 1.106 | | AcrA/AcrD/AcrF family protein |
| Smlt0049 | | 9.494 | | 66.154 | | Iron transporter protein |
| Smlt0050 | | 8.841 | | 28.724 | | Hypothetical protein |
| Smlt0051 | | 4.62 | | 21.318 | | Hypothetical protein |
| Smlt0052 | | 8.006 | | 39.016 | | Ku domain-containing protein |
| Smlt0073 | | 19.987 | | 79.869 | | Khg/kdpg aldolase |
| Smlt0084 | | 17.085 | | 63.444 | | Phosphatase |
| Smlt0085 | | 3.445 | | 30.216 | | Hypothetical protein |
| Smlt0086 | | 7.442 | | 97.258 | | Hypothetical protein |
| Smlt0089 | | 8.303 | | 30.757 | | Hypothetical protein |
| Smlt0107 | | 3.539 | | 50.685 | | Sensor histidine kinase transcriptional regulator |
| Smlt0108 | | 14.569 | | 78.033 | | Glutamate synthase subunit beta |
| Smlt0110 | | 5.486 | | 61.81 | | Viral-like DNA topoisomerase |
| Smlt0111 | | 16.115 | | 50.179 | | Hypothetical protein |
| Smlt0112 | | 7.578 | | 43.502 | | Putative short-chain dehydrogenase/reductase |
| Smlt0115 | | 10.743 | | 62.686 | | Putative beta-lactamase precursor |
| Smlt0117 | | 20.165 | | 114.078 | | Putative transmembrane BlaR protein |
| Smlt0119 | | 21.517 | | 77.779 | | Hypothetical protein |
| Smlt0123 | | 16.376 | | 89.723 | | Putative transmembrane protein |
| Smlt0124 | | 14.163 | | 58.265 | | Putative transmembrane protein |
| Smlt0125 | | 5.046 | | 48.117 | | Hypothetical protein |
| Smlt0126 | | 6.458 | | 56.703 | | Hypothetical protein |
| Smlt0127 | | 13.38 | | 84.856 | | Putative ATPase |
| Smlt0131 | | 20.492 | | 72.756 | | Putative peptidase/modulator of DNA gyrase |
| Smlt0157 | | 8.881 | | 31.478 | | Putative TonB dependent transport protein |
| Smlt0173 | | 16.763 | | 54.248 | | Hypothetical protein |
| Smlt0175 | | 14.844 | | 79.705 | | Hypothetical protein |
| Smlt0189 | | 12.669 | | 72.48 | | Putative transmembrane transporter protein |
| Smlt0224 | | 4.124 | | 25.631 | | Putative 3-dehydroquinate dehydratase |
| Smlt0225 | | 4.585 | | 62.183 | | Hypothetical protein |
| Smlt0231 | | 20.524 | | 67.491 | | Malate synthase |
| Smlt0244 | | 14.833 | | 100.809 | | Putative transmembrane protein |
| Smlt0272 | | 3.816 | | 14.256 | | Hypothetical protein |
| Smlt0279 | | 8.141 | | 31,732 | | Hypothetical protein |
| Smlt0284 | | 31.171 | | 121.863 | | Hypothetical protein |
| Smlt0343 | | 39.143 | | 192.024 | | Putative CsbD-like stress-like protein |
| Smlt0347 | | 43.747 | | 197.117 | | Metallo-beta-lactamase family protein |
| Smlt0349 | | 25.136 | | 77.109 | | Putative transmembrane protein |
| Smlt0358 | | 42.273 | | 160.356 | | Putative drug/metabolite transmembrane transporter protein |
| Smlt0361 | | 15.691 | | 53.839 | | Putative transmembrane permease |
| Smlt0362 | | 12.785 | | 68.247 | | Putative ATP-binding component of ABC transporter |
| Smlt0363 | | 20.054 | | 60.961 | | Putative HlyD family secretion protein |
| Smlt0364 | | 6.018 | | 52.543 | | Putative ATP binding component of ABC transporter |
| Smlt0365 | | 5.213 | | 58.15 | | Putative ABC transporter permease |
| Smlt0369 | | 0.377 | | 40.253 | | Putative transposase |
| Smlt0370 | | 11.831 | | 46.227 | | Hypothetical protein |
| Smlt0371 | | 33.59 | | 111.64 | | Putative cytochrome b-like protein |
| Smlt0372 | | 60.704 | | 375.484 | | Putative catalase |
| Smlt0377 | | 34.161 | | 186.744 | | Putative transport protein |
| Smlt0379 | | 139.212 | | 497.538 | | Putative transmembrane protein |
| Smlt0382 | | 7.2 | | 287.186 | | Hypothetical protein |
| Smlt0384 | | 8.195 | | 27.968 | | Hypothetical protein |
| Smlt0385 | | 11.338 | | 55.355 | | Hypothetical protein |
| Smlt0386 | | 18.817 | | 4790.318 | | Putative exported dipeptidyl peptidase IV |
| Smlt0391 | | 15.592 | | 56.701 | | Hypothetical protein |
| Smlt0392 | | 32.42 | | 112.796 | | Putative MFS family transporter |
| Smlt0395 | | 14.383 | | 116.19 | | Hypothetical protein |
| Smlt0399 | | 6.537 | | 43.795 | | DNA repair protein RadC |
| Smlt0400 | | 7.229 | | 112.567 | | Putative two-component response regulator transcriptional regulator |
| Smlt0405 | | 18.505 | | 78.397 | | Putative two-component sensor histidine kinase potassium transport regulator protein |
| Smlt0406 | | 6.472 | | 65.203 | | Putative potassium-transporting ATPase subunit C |
| Smlt0407 | | 13.627 | | 55.022 | | Potassium-transporting ATPase subunit B |
| Smlt0408 | | 13.012 | | 195.748 | | Potassium-transporting ATPase subunit A |
| Smlt0414 | | 37.618 | | 115.576 | | Hypothetical protein |
| Smlt0415 | | 50.141 | | 239.589 | | Anhydro-N-acetylmuramic acid kinase |
| Smlt0419 | | 35.746 | | 167.193 | | Hypothetical protein |
| Smlt0422 | | 41.418 | | 422.556 | | Putative rhomboid family transmembrane protein |
| Smlt0425 | | 11.401 | | 220.672 | | Putative transcriptional regulator NadR |
| Smlt0433 | | 24.048 | | 72.938 | | Putative transmembrane protein |
| Smlt0434 | | 7.376 | | 193.081 | | Hypothetical protein |
| Smlt0440 | | 34.427 | | 120.118 | | Hypothetical protein |
| Smlt0442 | | 28.163 | | 169.683 | | Putative ribonuclease |
| Smlt0443 | | 222.109 | | 1011.822 | | Hypothetical protein |
| Smlt0446 | | 49.122 | | 152.016 | | Hypothetical protein |
| Smlt0453 | | 12.191 | | 50.798 | | Hypothetical protein |
| Smlt0454 | | 16.079 | | 80.418 | | Putative transmembrane amino acid permease |
| Smlt0461 | | 13.367 | | 48.146 | | Putative TonB outer membrane protein oar family |
| Smlt0463 | | 22.693 | | 89.358 | | Putative D-alanyl-D-alanine dipeptidase |
| Smlt0467 | | 56.1 | | 304.768 | | Hypothetical protein |
| Smlt0480 | | 6.27 | | 93.668 | | Hypothetical protein |
| Smlt0481 | | 19.201 | | 114.554 | | Hypothetical protein |
| Smlt0482 | | 53.468 | | 213.313 | | Hypothetical protein |
| Smlt0485 | | 33.637 | | 174.028 | | Putative transmembrane protein |
| Smlt0499 | | 9.489 | | 72.265 | | Hypothetical protein |
| Smlt0500 | | 44.881 | | 170.001 | | Hypothetical protein |
| Smlt0505 | | 0.305 | | 50.665 | | Hypothetical protein |
| Smlt0508 | | 12.107 | | 69.08 | | Hypothetical protein |
| Smlt0518 | | 28.144 | | 113.414 | | Putative transposase |
| Smlt0519 | | 11.938 | | 39.667 | | Putative transmembrane protein |
| Smlt0520 | | 8.341 | | 82.478 | | Putative p47K/CobW family protein |
| Smlt0522 | | 13.02 | | 53.869 | | Putative LysR family transcriptional regulator |
| Smlt0523 | | 3.448 | | 96.978 | | Putative esterase |
| Smlt0525 | | 10.015 | | 46.746 | | Putative transmembrane EAL domain-containing protein |
| Smlt0531 | | 4.77 | | 32.831 | | Putative DGPF (potentially regulatory domain) protein |
| Smlt0532 | | 2.49 | | 28.932 | | Hypothetical protein |
| Smlt0534 | | 6.639 | | 66.248 | | Hypothetical protein |
| Smlt0544 | | 11.449 | | 55.321 | | Hypothetical protein |
| Smlt0548 | | 6.827 | | 34.566 | | Putative MFS family transporter |
| Smlt0553 | | 13.134 | | 71.505 | | Putative transmembrane protein |
| Smlt0554 | | 12.682 | | 98.054 | | Putative dioxygenase subunit |
| Smlt0558 | | 6.27 | | 54.72 | | Putative transposase-like protein |
| Smlt0559 | | 38.641 | | 209.093 | | Hypothetical protein |
| Smlt0561 | | 12.479 | | 49.337 | | Flagellar motor protein MotB |
| Smlt0566 | | 54.86 | | 426.068 | | Putative leucine-responsive regulatory protein |
| Smlt0572 | | 25.435 | | 139.2 | | Hypothetical protein |
| Smlt0578 | | 14.669 | | 56.342 | | DNA polymerase IV |
| Smlt0580 | | 17.902 | | 76.066 | | Putative metallo beta-lactamase family protein |
| Smlt0581 | | 13.805 | | 99.303 | | Putative metallo beta-lactamase family protein |
| Smlt0582 | | 50.117 | | 198.58 | | Putative LysR family transcriptional regulator |
| Smlt0592 | | 7.364 | | 54.549 | | Putative transmembrane protein |
| Smlt0593 | | 2.972 | | 58.829 | | Putative methionine sulfoxide reductase |
| Smlt0594 | | 20.298 | | 64.123 | | Putative two-component response regulator transcriptional regulator |
| Smlt0595 | | 12.719 | | 62.708 | | Putative sensor histidine kinase two-component transcriptional regulator |
| Smlt0596 | | 22.54 | | 169.316 | | Putative two component sensor histidine kinase transcriptional regulator |
| Smlt0599 | | 1.549 | | 37.412 | | Hypothetical protein |
| Smlt0600 | | 24.214 | | 131.51 | | Hypothetical protein |
| Smlt0603 | | 8.858 | | 280.722 | | Putative peptidase m14, carboxypeptidase A |
| Smlt0612 | | 97.814 | | 420.629 | | Putative twitching motility protein PilU protein |
| Smlt0614 | | 117.235 | | 4399.887 | | Putative outer membrane protein |
| Smlt0618 | | 0.792 | | 25.137 | | Putative transmembrane ABC transporter |
| Smlt0658 | | 25.975 | | 129.789 | | Hypothetical protein |
| Smlt0663 | | 48.172 | | 556.04 | | Putative transmembrane protein |
| Smlt0672 | | 28.031 | | 166.659 | | Putative transmembrane protein |
| Smlt0674 | | 78.813 | | 262.506 | | DNA polymerase III subunit chi |
| Smlt0682 | | 7.461 | | 146.515 | | Chitinase A |
| Smlt0685 | | 21.935 | | 79.323 | | Putative repetitive surface protein |
| Smlt0692 | | 25.792 | | 91.819 | | General secretion pathway protein J |
| Smlt0695 | | 23.618 | | 105.684 | | Putative general secretion pathway protein M |
| Smlt0696 | | 29.976 | | 95.232 | | Putative general secretion pathway protein N |
| Smlt0700 | | 27.125 | | 95.354 | | Hypothetical protein |
| Smlt0701 | | 40.025 | | 149.827 | | Putative polysaccharide production-like protein |
| Smlt0704 | | 13.089 | | 4229.202 | | Hypothetical protein |
| Smlt0715 | | 50.886 | | 475.009 | | Putative transmembrane permease |
| Smlt0722 | | 43.888 | | 161.125 | | Transcriptional regulator NrdR |
| Smlt0724 | | 1.228 | | 118.867 | | Putative transmembrane protein |
| Smlt0737 | | 6.251 | | 56.236 | | Putative O-antigen acetylase |
| Smlt0739 | | 8.869 | | 51.749 | | Putative transmembrane metal hydrolase protein |
| Smlt0741 | | 48.17 | | 214.723 | | Hypothetical protein |
| Smlt0746 | | 52.106 | | 668.457 | | Hypothetical protein |
| Smlt0758 | | 85.143 | | 332.439 | | Putative cell division protein FtsQ |
| Smlt0768 | | 5.737 | | 48.468 | | Putative alpha/beta hydrolase fold |
| Smlt0769 | | 12.754 | | 59.287 | | Putative transmembrane protein |
| Smlt0771 | | 11.796 | | 75.286 | | Putative membrane anchor amidohydrolase |
| Smlt0774 | | 67.221 | | 1256.088 | | Hypothetical protein |
| Smlt0779 | | 106.474 | | 600.271 | | Putative acyl-CoA thioester hydrolase |
| Smlt0781 | | 3.99 | | 22.539 | | Putative acyltransferase |
| Smlt0782 | | 3.781 | | 52.455 | | Hypothetical protein |
| Smlt0795 | | 19.976 | | 65.097 | | Putative exported heme receptor protein |
| Smlt0796 | | 2.853 | | 26.828 | | Hypothetical protein |
| Smlt0797 | | 9.576 | | 74.762 | | Hypothetical protein |
| Smlt0798 | | 8.945 | | 83.85 | | Putative inner membrane transport permease |
| Smlt0800 | | 53.686 | | 271.195 | | Putative acyl-CoA thioesterase I |
| Smlt0811 | | 21.545 | | 121.398 | | Putative transmembrane protein |
| Smlt0815 | | 21.845 | | 126.791 | | Hypothetical protein |
| Smlt0819 | | 55.833 | | 439.666 | | 4-hydroxythreonine-4-phosphate dehydrogenase |
| Smlt0830 | | 19.052 | | 63.438 | | Hypothetical protein |
| Smlt0831 | | 21.743 | | 213.691 | | Putative transmembrane protein |
| Smlt0833 | | 51.802 | | 290.838 | | Hypothetical protein |
| Smlt0840 | | 153.236 | | 1917.833 | | putative alkyl hydroperoxide reductase subunit |
| Smlt0842 | | 57.392 | | 283.609 | | HemK protein, putative protoporphyrinogen oxidase |
| Smlt0845 | | 7.516 | | 79.4 | | Putative transmembrane transport protein |
| Smlt0846 | | 47.353 | | 159.205 | | Hypothetical protein |
| Smlt0854 | | 20.535 | | 78.132 | | Putative transmembrane protein |
| Smlt0861 | | 44.946 | | 235.386 | | Putative secreted serine protease |
| Smlt0863 | | 20.377 | | 137.355 | | Putative transmembrane protein |
| Smlt0865 | | 49.155 | | 203.134 | | Ribonuclease BN/unknown domain fusion protein |
| Smlt0869 | | 45.963 | | 142.016 | | Peptide chain release factor 1 |
| Smlt0875 | | 255.785 | | 1690.144 | | Ribose-phosphate pyrophosphokinase |
| Smlt0939 | | 12.147 | | 41.699 | | Putative transmembrane protein |
| Smlt0952 | | 23.338 | | 80.258 | | Putative 2,4-dienoyl-CoA reductase [NADPH] |
| Smlt0972 | | 4.389 | | 36.326 | | Putative transmembrane PAP family protein |
| Smlt0973 | | 16.314 | | 49.988 | | Putative mannosyltransferase PimB |
| Smlt0974 | | 6.212 | | 44.096 | | Putative TonB dependent receptor protein |
| Smlt1019 | | 20.459 | | 77.004 | | Putative transmembrane transglutaminase |
| Smlt1046 | | 1.197 | | 6.575 | | Hypothetical protein |
| Smlt1048 | | 4.726 | | 50.52 | | Putative bacteriophage tail protein I |
| Smlt1049 | | 3.67 | | 16.837 | | Putative phage baseplate assembly protein |
| Smlt1050 | | 0.777 | | 28.164 | | Putative phage baseplate assembly protein |
| Smlt1051 | | 2.896 | | 21.736 | | Putative baseplate assembly protein |
| Smlt1052 | | 4.982 | | 34.719 | | Putative phage-like protein |
| Smlt1053 | | 3.831 | | 55.568 | | Hypothetical protein |
| Smlt1054 | | 2.167 | | 21.074 | | Putative endolysin/phage lysozyme |
| Smlt1055 | | 2.626 | | 12.364 | | Hypothetical protein |
| Smlt1056 | | 5.852 | | 44.845 | | Hypothetical protein |
| Smlt1068 | | 10.229 | | 41.612 | | Putative transmembrane protein |
| Smlt1078 | | 8.059 | | 45.506 | | Putative major facilitator superfamily transmembrane transporter protein |
| Smlt1083 | | 18.796 | | 56.399 | | Putative major facilitator superfamily transmembrane transporter protein |
| Smlt1123 | | 21.745 | | 79.958 | | Phosphoribosylglycinamide formyltransferase |
| Smlt1138 | | 13.043 | | 48.779 | | Putative oxidoreductase |
| Smlt1144c | | 7.396 | | 27.032 | | Putative exogenous ferric siderophore receptor |
| Smlt1147 | | 22.858 | | 74.5 | | Hypothetical protein |
| Smlt1152 | | 5.056 | | 29.599 | | Hypothetical protein |
| Smlt1156 | | 36.736 | | 114.014 | | Hypothetical protein |
| Smlt1170 | | 33.175 | | 118.807 | | UDP-N-acetylmuramoyl-L-alanyl-D-glutamate synthetase |
| Smlt1174 | | 13.931 | | 49.583 | | Putative alpha-glucosidase AglA |
| Smlt1175 | | 6.433 | | 37.413 | | Putative TonB dependent receptor protein |
| Smlt1176 | | 2.117 | | 26.31 | | Putative alpha-glucosidase protein |
| Smlt1177 | | 14.921 | | 45.953 | | Hypothetical protein |
| Smlt1178 | | 13.184 | | 41.415 | | Putative major facilitator superfamily transmembrane transporter protein |
| Smlt1184 | | 5.839 | | 36.413 | | Putative transmembrane protein |
| Smlt1185 | | 4.957 | | 17.302 | | Putative ABC transporter ATP-binding protein |
| Smlt1198 | | 11.628 | | 44.841 | | Putative transmembrane GGDEF domain signalling protein |
| Smlt1201 | | 8.31 | | 26.364 | | Putative thioesterase |
| Smlt1202 | | 8.172 | | 31.925 | | Putative transmembrane protein |
| Smlt1208 | | 3.511 | | 16.202 | | Putative DNA-binding protein |
| Smlt1212 | | 4.299 | | 22.91 | | Hypothetical protein |
| Smlt1213 | | 3.424 | | 21.34 | | Hypothetical protein |
| Smlt1214 | | 6.073 | | 50.222 | | Hypothetical protein |
| Smlt1215 | | 2.245 | | 28.259 | | Putative transmembrane DoxX family protein |
| Smlt1216 | | 5.426 | | 22.094 | | Hypothetical protein |
| Smlt1217 | | 4.099 | | 13.989 | | Putative exported thioredoxin |
| Smlt1219 | | 12.124 | | 49.277 | | Putative transmembane sensor histidine kinase transcriptional regulator |
| Smlt1220 | | 20.386 | | 56.324 | | Hypothetical protein |
| Smlt1221 | | 19.541 | | 75.637 | | Putative major facilitator superfamily transmembrane transporter protein |
| Smlt1226 | | 6.719 | | 52.775 | | Putative malonyl CoA-acyl carrier protein transacylase |
| Smlt1227 | | 14.321 | | 60.301 | | Putative coenzyme-A synthase |
| Smlt1233 | | 8.945 | | 42.848 | | Putative TonB-dependent receptor for Fe(III)-coprogen, Fe(III)-ferrioxamine B and Fe(III)-rhodotrulic acid |
| Smlt1242 | | 3.438 | | 37.922 | | Putative transmembrane protein |
| Smlt1268 | | 14.551 | | 61.887 | | Putative calcium-binding protein |
| Smlt1283 | | 3.448 | | 23.008 | | Putative conjugal transfer protein |
| Smlt1284 | | 4.002 | | 17.764 | | Putative conjugal transfer protein |
| Smlt1285 | | 2.054 | | 14.035 | | Conjugal transfer protein TrbF |
| Smlt1286 | | 0.811 | | 10.29 | | Conjugal transfer protein TrbL |
| Smlt1287 | | 1.962 | | 20.935 | | Conjugal transfer protein TrbJ |
| Smlt1288 | | 3.706 | | 22.476 | | Conjugal transfer ATPase TrbE |
| Smlt1289 | | 0.482 | | 17.169 | | Putative conjugal transfer protein |
| Smlt1290 | | 0.686 | | 23.507 | | Putative conjugal transfer protein |
| Smlt1291 | | 0.506 | | 22.679 | | Putative conjugal transfer protein |
| Smlt1292 | | 0.295 | | 19.806 | | Hypothetical protein |
| Smlt1293 | | 1.184 | | 28.976 | | Conjugal transfer coupling protein TraG |
| Smlt1308 | | 4.824 | | 26.856 | | Hypothetical protein |
| Smlt1310 | | 3.429 | | 22.302 | | Putative transmembrane anchor conjugal transfer protein |
| Smlt1311 | | 0.502 | | 35.049 | | Hypothetical protein |
| Smlt1312 | | 1.995 | | 10.521 | | Putative parB partition protein |
| Smlt1313 | | 2.885 | | 38.576 | | Putative ParA/CobQ/CobB/MinD nucleotide binding domain-containing protein |
| Smlt1314 | | 0.947 | | 9.158 | | Putative RepA-like replication protein |
| Smlt1315 | | 2.262 | | 13.72 | | Hypothetical protein |
| Smlt1320A | | 3.424 | | 38.166 | | Hypothetical protein |
| Smlt1325 | | 3.483 | | 36.739 | | Hypothetical protein |
| Smlt1326 | | 1.491 | | 14.616 | | Putative ParB-like nuclease domain-containing protein |
| Smlt1327 | | 2.535 | | 14.832 | | Hypothetical protein |
| Smlt1344 | | 9.885 | | 47.178 | | Putative transmembrane protein |
| Smlt1365 | | 11.836 | | 53.816 | | Putative transmembrane GGDEF EAL signalling protein |
| Smlt1402 | | 12.438 | | 38.654 | | Hypothetical protein |
| Smlt1408 | | 17.027 | | 63.67 | | Putative transmembrane LysE family transport protein |
| Smlt1417 | | 8.865 | | 37.662 | | Putative nucleotide sugar transaminase |
| Smlt1425 | | 10.926 | | 38.176 | | Putative GGDEF family signalling protein |
| Smlt1452 | | 22.879 | | 82.982 | | Putative ADA-like transcriptional regulator |
| Smlt1453 | | 3.442 | | 18.721 | | Hypothetical protein |
| Smlt1468 | | 6.591 | | 41.332 | | Hypothetical protein |
| Smlt1473 | | 5.42 | | 23.355 | | Poly(beta-D-mannuronate) lyase |
| Smlt1478 | | 11.703 | | 54.007 | | Putative LysR family transcriptional regulator |
| Smlt1479 | | 9.174 | | 37.595 | | N-terminus of bpertussis putative membrane protein BP0712 |
| Smlt1484 | | 9.353 | | 38.418 | | Putative PadR family transcriptional regulator |
| Smlt1510 | | 8.245 | | 72.749 | | Putative exported lipoprotein |
| Smlt1511 | | 4.754 | | 38.916 | | Putative fimbriae usher protein |
| Smlt1512 | | 5.42 | | 34.58 | | Putative exported fimbriae-related chaperone |
| Smlt1513 | | 8.926 | | 62.768 | | Hypothetical protein |
| Smlt1537 | | 12.973 | | 52.163 | | Putative outer membrane efflux protein |
| Smlt1538 | | 11.829 | | 37.217 | | Putative macrolide-specific ABC-type efflux carrier |
| Smlt1544 | | 4.036 | | 12.637 | | Putative transmembrane protein |
| Smlt1545 | | 7.489 | | 34.902 | | Hypothetical protein |
| Smlt1546 | | 4.509 | | 25.96 | | Hypothetical protein |
| Smlt1557 | | 27.523 | | 82.873 | | Hypothetical protein |
| Smlt1565 | | 11.969 | | 41.031 | | Putative transmembrane protein |
| Smlt1571a | | 13.72 | | 46.964 | | Putative aldehyde dehydrogenase |
| Smlt1637 | | 16.129 | | 93.668 | | Putative transmembrane protein |
| Smlt1648 | | 5.709 | | 43.751 | | Putative FAD binding protein |
| Smlt1659 | | 8.44 | | 48.22 | | Hypothetical protein |
| Smlt1660 | | 2.926 | | 24.689 | | Putative modification methylase |
| Smlt1750 | | 3.151 | | 24.333 | | Putative ECF family sigma facto |
| Smlt1751 | | 3.218 | | 46.325 | | Putative transmembrane FecR sensor protein |
| Smlt1753 | | 9.818 | | 41.61 | | Putative TonB dependent receptor protein |
| Smlt1754 | | 10.972 | | 36.328 | | Putative alkaline phosphatase |
| Smlt1760 | | 16.054 | | 71.874 | | Putative transmembrane major facilitator superfamily transporter protein |
| Smlt1810 | | 26.833 | | 88.445 | | Hypothetical protein |
| Smlt1822 | | 9.817 | | 41.114 | | Putative phosphoesterase |
| Smlt1829 | | 8.709 | | 49.309 | | Putative short chain dehydrogenase |
| Smlt1830 | | 13.323 | | 47.531 | | Putative multidrug efflux protein, HlyD family |
| Smlt1831 | | 4.775 | | 23.43 | | Putative drug resistance membrane fusion protein |
| Smlt1832 | | 3.728 | | 17.203 | | Putative short-chain dehydrogenase/reductase |
| Smlt1833 | | 7.145 | | 30.461 | | Putative outer membrane efflux protein |
| Smlt1987 | | 7.488 | | 38.256 | | Putative GDSL-like lipase/acylhydrolase |
| Smlt1995 | | 8.229 | | 33.995 | | Putative major facilitator superfamily transmembrane protein |
| Smlt2029 | | 20.441 | | 65.791 | | Putative LysR family transcriptional regulator |
| Smlt2033 | | 14.401 | | 46.713 | | Putative HlyD family secretion protein |
| Smlt2035 | 10.023 | | | 30.197 | | Putative transmembrane ACR-type efflux transport protein |
| Smlt2061 | 6.144 | | | 29.858 | | Putative LysE family transmembrane transporter |
| Smlt2062 | 2.898 | | | 11.737 | | Putative amino acid LysE family efflux protein |
| Smlt2081 | 7.837 | | | 30.999 | | Putative ThiJ/PfpI family protein |
| Smlt2098 | 6.964 | | | 33.956 | | Putative AraC family regulatory protein |
| Smlt2099 | 4.343 | | | 21.084 | | Putative TonB dependent receptor protein |
| Smlt2100 | 2.036 | | | 30.424 | | Putative NAD(P)H-dependent FMN reductase |
| Smlt2101 | 4.389 | | | 22.256 | | Hypothetical protein |
| Smlt2102 | 1.75 | | | 38.517 | | Hypothetical protein |
| Smlt2103 | 3.527 | | | 19.762 | | Putative monooxygenase |
| Smlt2104 | 3.343 | | | 26.728 | | Putative amidohydrolase |
| Smlt2105 | 2.147 | | | 17.925 | | Putative acetyltransferase |
| Smlt2106 | 3.318 | | | 34.997 | | Putative transmembrane ABC transporter |
| Smlt2107 | 3.5 | | | 43.132 | | Putative substrate-binding ABC transport protein |
| Smlt2108 | 6.963 | | | 25.483 | | Putative exported 5/8 type C domain-containing protein |
| Smlt2110 | 5.816 | | | 18.475 | | Putative DNA-binding protein |
| Smlt2134 | 6.796 | | | 52.604 | | Putative acetyltransferase |
| Smlt2135 | 23.939 | | | 91.974 | | Putative acetyltransferase |
| Smlt2136 | 94.86 | | | 431.162 | | Putative AraC family regulatory protein |
| Smlt2149 | 7.816 | | | 50.73 | | Putative ArsR family regulatory protein |
| Smlt2212 | 14.457 | | | 43.568 | | Hypothetical protein |
| Smlt2225 | 0.096 | | | 1.653 | | Putative transmembrane protein |
| Smlt2247 | 8.746 | | | 32.053 | | Hypothetical protein |
| Smlt2250 | 4.691 | | | 19.554 | | Putative chemotaxis protein methyltransferase |
| Smlt2254 | 12.452 | | | 39.695 | | Putative methyl-accepting chemotaxis protein |
| Smlt2258 | 6.964 | | | 32.173 | | Putative methyl-accepting chemotaxis receptor |
| Smlt2263 | 10.728 | | | 52.292 | | Putative protein with a CheW-like domain |
| Smlt2264 | 10.93 | | | 49.218 | | Putative ParA-like protein |
| Smlt2265 | 10.098 | | | 39.259 | | Flagellar motor protein MotD |
| Smlt2267 | 9.929 | | | 46.197 | | Putative chemotaxis two component regulator sensor histidine kinase transcriptional regulator |
| Smlt2268 | 16.947 | | | 64.453 | | Putative chemotaxis protein |
| Smlt2270 | 8.141 | | 43.398 | | Putative RNA polymerase sigma factor for flagellar regulon FliA | |
| Smlt2272 | 7.788 | | 73.373 | | Flagellar biosynthesis regulator FlhF | |
| Smlt2273 | 4.932 | | 32.43 | | Flagellar biosynthesis protein FlhA | |
| Smlt2274 | 6.636 | | 28.548 | | Flagellar biosynthesis protein FlhB | |
| Smlt2277 | 3.742 | | 22.428 | | Putative flagellar biosynthetic protein | |
| Smlt2278 | 1.951 | | 21.86 | | Putative flagellar biosynthetic protein FliQ | |
| Smlt2280 | 4.702 | | 45.878 | | Putative flagellar protein FliO | |
| Smlt2281 | 3.527 | | 14.983 | | Putative flagellar motor switch protein | |
| Smlt2282 | 6.157 | | 21.937 | | Flagellar motor switch protein FliM | |
| Smlt2283 | 4.848 | | 16.821 | | Putative flagellar basal body-associated protein FliL | |
| Smlt2284 | 11.311 | | 59.356 | | Putative flagellar hook-length control protein | |
| Smlt2286 | 6.555 | | 48.972 | | Putative flagellum-specific ATP synthase | |
| Smlt2287 | 5.537 | | 27.039 | | Putative flagellar assembly protein, FliH | |
| Smlt2288 | 6.833 | | 24.428 | | Putative flagellar motor switch protei | |
| Smlt2289 | 5.436 | | 29.723 | | Flagellar MS-ring protein | |
| Smlt2290 | 6.475 | | 22.292 | | Putative flagellar hook-basal body complex protein | |
| Smlt2293 | 11.703 | | 48.606 | | Putative transmembrane protein | |
| Smlt2294 | 6.309 | | 21.699 | | Hypothetical protein | |
| Smlt2308 | 13.929 | | 45.036 | | Flagellar hook-associated protein FlgK | |
| Smlt2309 | 8.969 | | 44.147 | | Flagellar rod assembly protein/muramidase FlgJ | |
| Smlt2310 | 10.137 | | 35.539 | | Flagellar basal body P-ring protein | |
| Smlt2311 | 10.83 | | 43.085 | | Flagellar basal body L-ring protein | |
| Smlt2312 | 11.893 | | 36.441 | | Flagellar basal body rod protein FlgG | |
| Smlt2313 | 8.075 | | 24.534 | | Flagellar basal body rod protein FlgF | |
| Smlt2317 | 7.98 | | 24.11 | | Flagellar basal body rod protein FlgB | |
| Smlt2325 | 9.207 | | 28.24 | | Putative PAS GGDEF EAL domain-containing protein | |
| Smlt2355 | 8.977 | | 39.453 | | Putative binding-protein-dependent transport lipoprotein | |
| Smlt2356 | 3.531 | | 36.248 | | Putative FecCD family transmembrane transport protein | |
| Smlt2357 | 1.901 | | 30.071 | | Putative ABC transporter ATP-binding protein | |
| Smlt2369 | 4.69 | | 45.055 | | Putative transmembrane protein | |
| Smlt2370 | 4.099 | | 29.886 | | Hypothetical protein | |
| Smlt2372 | 6.515 | | 25.768 | | Hypothetical protein | |
| Smlt2373 | 4.121 | | 18.337 | | Putative amine oxidoreductase | |
| Smlt2374 | 6.507 | | 34.317 | | Putative fatty acid desaturase (membrane) | |
| Smlt2375 | 2.057 | | 29.836 | | Hypothetical protein | |
| Smlt2378 | 6.437 | | 21.86 | | Putative transcriptional regulator | |
| Smlt2412 | 8.312 | | 34.719 | | Putative mercuric reductase | |
| Smlt2497 | 9.753 | | 30.31 | | Putative transmembrane protein | |
| Smlt2499 | 10.733 | | 40.568 | | Hypothetical protein | |
| Smlt2500 | 5.311 | | 21.008 | | Hypothetical protein | |
| Smlt2507 | 6.379 | | 21.531 | | Putative organic hydroperoxide resistance protein | |
| Smlt2508 | 7.715 | | 28.48 | | Hypothetical protein | |
| Smlt2509 | 2.296 | | 22.877 | | Putative LysR family transcriptional regulator | |
| Smlt2510 | 2.557 | | 7.865 | | Putative transcriptional regulator sigma | |
| Smlt2511 | 3.017 | | 20.614 | | Hypothetical protein | |
| Smlt2512 | 1.625 | | 12.43 | | Putative cupin domain-containing protein | |
| Smlt2514 | 9.149 | | 31.665 | | Putative beta-lactamase | |
| Smlt2516 | 13.663 | | 42.034 | | Putative esterase/chloroperoxidase | |
| Smlt2526 | 8.404 | | 25.444 | | Putative transmembrane protein | |
| Smlt2527 | 5.725 | | 18.068 | | Putative iron-sulfur binding oxidoreductase | |
| Smlt2532 | 5.288 | | 33.115 | | Hypothetical protein | |
| Smlt2533 | 5.732 | | 17.95 | | Putative DNA-formamidopyrimidine glycosylase | |
| Smlt2535 | 7.315 | | 26.707 | | Putative sensors of blue light using FAD protein | |
| Smlt2537 | 4.49 | | 22.917 | | Putative manganese containing catalase | |
| Smlt2540 | 3.35 | | 22.086 | | Putative two component response regulator transcriptional regulator | |
| Smlt2541 | 2.97 | | 9.572 | | Hypothetical protein | |
| Smlt2545 | 2.602 | | 17.611 | | Hypothetical protein | |
| Smlt2546 | 2.935 | | 32.065 | | Hypothetical protein | |
| Smlt2550 | 3.884 | | 11.778 | | Putative transmembrane protein | |
| Smlt2551 | 6.124 | | 18.84 | | Putative transmembrane protein | |
| Smlt2552 | 5.541 | | 20.749 | | Putative isochorismatase | |
| Smlt2571 | 7.223 | | 44.604 | | Putative phosphate selective porin | |
| Smlt2572 | 8.134 | | 28.15 | | Putative ABC transporter transmembrane permease | |
| Smlt2575 | 7.393 | | 31.872 | | Hypothetical protein | |
| Smlt2603 | 7.466 | | 30.329 | | Putative hexuronate transporter | |
| Smlt2604 | 12.065 | | 48.873 | | Putative 3-oxoacyl-[acyl carrier protein] reductase | |
| Smlt2605 | 10.085 | | 32.585 | | Putative GDSL-like lipase/acylhydrolase exported protein | |
| Smlt2606 | 6.712 | | 34.378 | | Putative PfkB family carbohydrate kinase | |
| Smlt2611 | 8.408 | | 28.6 | | Putative endonuclease | |
| Smlt2617 | 5.692 | | 19.177 | | Hypothetical protein | |
| Smlt2622 | 3.048 | | 28.128 | | Hypothetical protein | |
| Smlt2623 | 2.13 | | 41.292 | | Hypothetical protein | |
| Smlt2624 | 7.398 | | 27.285 | | Putative YadA/Hep-Hag like protein | |
| Smlt2625 | 4.295 | | 15.513 | | Putative peptidase protein | |
| Smlt2626 | 6.096 | | 19.518 | | Hypothetical protein | |
| Smlt2633 | 9.056 | | 41.791 | | Putative DNA glycosylase | |
| Smlt2634 | 0.665 | | 2.63 | | Hypothetical protein | |
| Smlt2637 | 7.052 | | 31.284 | | Putative trehalose synthase | |
| Smlt2638 | 1.868 | | 28.317 | | Putative dehydrogenase | |
| Smlt2640 | 4.252 | | 24.427 | | Putative dehydrogenase/oxidoreductase protein | |
| Smlt2663 | 3.027 | | 51.213 | | Putative transmembrane protein | |
| Smlt2675 | 11.033 | | 65.623 | | Putative carboxymuconolactone decarboxylase family protein | |
| Smlt2678 | 4.896 | | 28.309 | | Putative transmembrane protein | |
| Smlt2679 | 6.548 | | 26.366 | | Hypothetical protein | |
| Smlt2680 | 2.968 | | 23.481 | | Putative lipoprotein | |
| Smlt2681 | 2.992 | | 24.658 | | Putative lipoprotein | |
| Smlt2682 | 3.649 | | 32.025 | | Putative ABC transporter transmembrane permease | |
| Smlt2683 | 4.54 | | 16.96 | | Putative ABC-transporter ATP binding protein | |
| Smlt2684 | 3.452 | | 25.031 | | Hypothetical protein | |
| Smlt2687 | 3.111 | | 20.08 | | 2,5-diketo-D-gluconate reductase B | |
| Smlt2688 | 6.528 | | 40.835 | | Putative monooxygenase | |
| Smlt2696 | 4.424 | | 29.632 | | Putative short chain dehydrogenease | |
| Smlt2697 | 7.144 | | 30.681 | | Putative cation efflux-related membrane protein | |
| Smlt2698 | 6.174 | | 49.725 | | Putative cobalt-zinc-cadmium resistance protein | |
| Smlt2712 | 8.028 | | 28.227 | | hypothetical protein | |
| Smlt2713 | 8.95 | | 28.592 | | hypothetical protein | |
| Smlt2714 | 7.589 | | 34.352 | | putative TonB dependent protein | |

| Smlt2715 | 11.1 | 47.94 | Putative transmembrane sensor histidine kinase transcriptional regulator |
| --- | --- | --- | --- |
| Smlt2723 | 12.368 | 55.76 | Hypothetical protein |
| Smlt2724 | 5.767 | 29.623 | Hypothetical protein |
| Smlt2725 | 6.002 | 50.446 | Putative proline-rich protein |
| Smlt2727 | 2.139 | 38.414 | Putative transmembrane MotA/TolQ/ExbB transport protein |
| Smlt2728 | 1.377 | 30.854 | Putative exported surface antigen protein |
| Smlt2729 | 3.281 | 17.846 | Hypothetical protein |
| Smlt2730 | 9.12 | 34.193 | Putative general secretory pathway protein |
| Smlt2731 | 2.356 | 46.602 | Putative general secretion pathway protein H |
| Smlt2732 | 3.264 | 18.65 | Putative general secretion pathway protein I |
| Smlt2733 | 6.637 | 22.299 | Putative general secretion pathway protein J |
| Smlt2738 | 2.463 | 15.499 | Putative alkaline phosphatase l |
| Smlt2740 | 5.596 | 48.461 | Putative general secretion pathway protein F |
| Smlt2741 | 5.325 | 32.443 | Putative general secretion pathway protein E |
| Smlt2742 | 3.542 | 35.025 | Putative general secretion pathway protein D |
| Smlt2743 | 9.725 | 46.028 | Putative general secretion pathway protein M |
| Smlt2744 | 3.393 | 41.459 | Putative general secretion pathway protein L |
| Smlt2745 | 2.471 | 35.255 | Putative general secretion pathway protein K |
| Smlt2747 | 10.353 | 32.048 | Hypothetical protein |
| Smlt2749 | 5.503 | 44.239 | Putative signal transduction protein FecR |
| Smlt2750 | 2.232 | 29.749 | Putative sigma 70 subunit of RNA polymerase |
| Smlt2751 | 4.64 | 41.805 | Putative TonB dependent family protein |
| Smlt2754 | 8.993 | 27.035 | Putative transmembrane AzlC amino acid transport protein |
| Smlt2755 | 7.942 | 29.208 | Putative transmembrane amino acid transporter protein |
| Smlt2756 | 8.753 | 31.265 | Putative glycogen debranching enzyme |
| Smlt2757 | 4.348 | 35.002 | Putative alpha amylase/glycosyl hydrolase |
| Smlt2758 | 3.425 | 42.246 | Putative 4-alpha-glucanotransferase |
| Smlt2759 | 6.905 | 34.698 | Putative Maltooligosyltrehalose trehalohydrolase protein |
| Smlt2760 | 5.53 | 29.785 | Glycogen branching enzyme |
| Smlt2761 | 4.397 | 32.741 | Glycogen synthase |
| Smlt2763 | 9.208 | 28.478 | Putative sulphite reductase flavodoxin containing alpha subunit |
| Smlt2796 | 3.206 | 16.47 | Putative transmembrane fusaric acid resistance efflux protein |
| Smlt2797 | 8.034 | 30.011 | Putative multidrug resistance protein |
| Smlt2798 | 13.56 | 41.918 | Putative outer membrane multidrug efflux protein |
| Smlt2806 | 30.173 | 94.546 | Hypothetical protein |
| Smlt2817 | 6.939 | 38.424 | Putative 2,3-dihydro-2,3-dihydroxybenzoate dehydrogenase |
| Smlt2818 | 5.211 | 41.223 | Enterobactin synthase subunit F |
| Smlt2820 | 2.912 | 14.809 | Putative hydrolase |
| Smlt2821 | 7.328 | 24.364 | Putative siderophore specific 2,3-dihydroxybenzoate-AMP ligase |
| Smlt2822 | 5.624 | 59.318 | Putative isochorismate synthase |
| Smlt2827 | 18.667 | 59.724 | Hypothetical protein |
| Smlt2859 | 16.634 | 54.438 | Putative transporter transmembrane protein |
| Smlt2864 | 7.29 | 46.33 | Putative OmpA family protein |
| Smlt2865 | 19.405 | 64.457 | Putative haemagglutinin-like protein |
| Smlt2866 | 8.348 | 30.662 | Putative AraC family transcriptional regulator |
| Smlt2868 | 6.475 | 24.663 | Putative transmembrane peptidase |
| Smlt2869 | 4.416 | 19.288 | Putative TadE family transmembrane pilus-like protein |
| Smlt2870 | 8.372 | 32.779 | Putative transmembrane fimbriae assembly protein |
| Smlt2872 | 7.123 | 42.121 | Putative fimbriae assembly protein |
| Smlt2875 | 7.315 | 22.997 | Putative transmembrane type II secretion protein |
| Smlt2876 | 7.241 | 56.501 | TPR repeat-containing protein |
| Smlt2877 | 6.729 | 60.565 | Hypothetical protein |
| Smlt2882 | 4.628 | 17.675 | Putative non-heme chloroperoxidase |
| Smlt2883 | 2.708 | 24.364 | Putative major facilitator superfamily transmembrane transporter |
| Smlt2884 | 3.794 | 22.713 | Hypothetical protein |
| Smlt2885 | 3.349 | 20.854 | Putative amidohydrolase protein |
| Smlt2886 | 3.091 | 30.562 | Putative transmembrane protein |
| Smlt2888 | 0.593 | 28.932 | Putative AraC family transcriptional regulator |
| Smlt2889 | 0.791 | 23.98 | Putative transmembrane protein |
| Smlt2890 | 9.56 | 41.364 | Putative two-component system sensor histidine kinase |
| Smlt2892 | 12.603 | 65.152 | Putative major facilitator superfamily transmembrane transporter |
| Smlt2897 | 14.128 | 42.407 | Hypothetical protein |
| Smlt2902 | 3.756 | 26.586 | Hypothetical protein |
| Smlt2918 | 5.135 | 42.408 | Putative LysR family transcriptional regulator |
| Smlt2917 | 15.412 | 56.316 | Putative major facilitator superfamily transmembrane transport protein |
| Smlt2924 | 5.258 | 22.132 | Putative thioredoxin DsbA family |
| Smlt2926 | 8.349 | 65.485 | Hypothetical protein |
| Smlt2927 | 6.443 | 53.802 | Hypothetical protein |
| Smt2928 | 6.01 | 32.754 | Error-prone DNA polymerase |
| Smlt2935 | 6.712 | 26.209 | Putative ECF family sigma factor |
| Smlt2936 | 4.119 | 26.779 | Putative FecR iron transport regulator family protein |
| Smlt2937 | 9.552 | 36.42 | Putative TonB dependent receptor protein |
| Smlt2938 | 18.41 | 61.549 | Putative iron regulated lipoprotein |
| Smlt2970 | 9.453 | 52.523 | Putative transmembrane drug/sodium antiporter |
| Smlt2978 | 11.208 | 34.532 | Putative NADH:flavin oxidoreductase/NADH oxidase |
| Smlt3019 | 15.067 | 49.714 | Putative two-component sensor histidine kinase/response regulator fusion protein |
| Smlt3020 | 27.041 | 190.565 | Putative FAD binding monooxygenase |
| Smlt3021 | 61.239 | 298.741 | Putative TetR family transcriptional regulator |
| Smlt3136 | 11.922 | 44.891 | Putative transmembrane quinoprotein glucose dehydrogenase |
| Smlt3202 | 4.223 | 36.356 | Putative exported alkaline phosphatase D |
| Smlt3203 | 7.964 | 27.514 | Putative TonB-dependent receptor |
| Smlt3223 | 7.633 | 77.217 | Putative ECF-sigma factor |
| Smlt3224 | 8.719 | 29.89 | Putative carboxymuconolactone family protein |
| Smlt3262 | 13.35 | 46.864 | Putative AraC family transcriptional regulator |
| Smlt3263 | 11.711 | 40.396 | Putative transmembrane major facilitator superfamily transport protein |
| Smlt3289 | 6.492 | 29.874 | Putative haemin storage system, HmsH protein |
| Smlt3290 | 5.582 | 33.302 | Putative hemin storage protein |
| Smlt3291 | 3.214 | 25.026 | N-glycosyltransferase |
| Smlt3294 | 13.91 | 79.682 | Gamma-glutamyl kinase |
| Smlt3295 | 12.967 | 48.659 | Putative YceI domain-containing protein |
| Smlt3303 | 4.876 | 28.932 | Putative transmembrane protein |
| Smlt3345 | 12.786 | 52.852 | Short chain dehydrogenase |
| Smlt3379 | 14.186 | 67.216 | Putative transmembrane protein |
| Smlt3380 | 7.98 | 51.289 | Putative transmembrane protein |
| Smlt3418 | 25.134 | 116.726 | Hypothetical protein |
| Smlt3425 | 45.339 | 147.59 | Putative glyoxalase/bleomycin resistance protein |
| Smlt3426 | 99.159 | 326.942 | Putative fimV protein |
| Smlt3437 | 10.382 | 69.59 | Hypothetical protein |
| Smlt3439 | 22.924 | 104.648 | Putative carboxymethylenebutenolidase |
| Smlt3441 | 51.725 | 250.543 | Putative transmembrane protein |
| Smlt3457 | 14.419 | 50.094 | Putative D-amino acid dehydrogenase small subunit |
| Smlt3496 | 15.93 | 49.72 | Putative glyoxalase/bleomycin resistance protein |
| Smlt3509 | 15.564 | 54.933 | Putative heat shock chaperone protein |
| Smlt3513 | 27.615 | 84.846 | Hypothetical protein |
| Smlt3522 | 11.125 | 49.322 | Hypothetical protein |
| Smlt3529 | 4.217 | 31.452 | Hypothetical protein |
| Smlt3530 | 9.007 | 37.029 | ATP-dependent DNA ligase |
| Smlt3531 | 7.868 | 49.82 | Putative ATP-dependent DNA helicase |
| Smlt3571 | 8.618 | 40.4 | Putative transmembrane sulfatase |
| Smlt3585 | 14.748 | 48.962 | Hypothetical protein |
| Smlt3590 | 10.681 | 52.488 | Putative endopeptidase/peptidoglycan hydrolase |
| Smlt3602 | 14.281 | 51.129 | Putative penicillin-binding protein |
| Smlt3636 | 20.302 | 64.162 | Putative peptidase family protein |
| Smlt3656 | 5.852 | 32.575 | Putative ABC transporter protein |
| Smlt3691b2 | 14.993 | 64.294 | Putative copper resistance protein/multicopper oxidase |
| Smlt3730 | 14.799 | 47.368 | Putative transmembrane two component system sensor histidine kinase protein |
| Smlt3745 | 17.891 | 57.127 | Hypothetical protein |
| Smlt3778 | 1.965 | 6.046 | Hypothetical protein |
| Smlt3786 | 13.954 | 48.072 | Putative acetyltransferase |
| Smlt3687 | 11.414 | 41.76 | Putative transmembrane protein |
| Smlt3793 | 23.951 | 73.565 | Transcriptional regulator CysB-like protein |
| Smlt3811 | 13.921 | 44.798 | Putative flavoredoxin |
| Smlt3830 | 0.186 | 4.413 | Putative giant cable pilus chaperone protein |
| Smlt3831 | 0.115 | 8.18 | Putative minor pilin and initiator protein |
| Smlt3832a | 1.014 | 21.58 | Putative outer membrane usher |
| Smlt3834 | 5.441 | 39.214 | Transmembrane protein |
| Smlt3892 | 15.544 | 79.322 | Putative TonB dependent receptor protein |
| Smlt3893 | 8.345 | 44.009 | Putative ExbD/TolR family transport protein |
| Smlt3894 | 17.874 | 63.231 | Putative MotA/TolQ/ExbB proton channel family protein |
| Smlt3895 | 7.315 | 40.431 | Putative transmembrane protein |
| Smlt3896 | 12.539 | 40.446 | Putative iron-starvation protein PigA |
| Smlt3899 | 22.218 | 71.245 | Putative transmembrane FecR sensor protein |
| Smlt3900A | 3.211 | 28.932 | Hypothetical protein |
| Smlt3911 | 0.482 | 1.908 | Hypothetical protein |
| Smlt3912 | 12.387 | 37.314 | Putative glutamyltransferase |
| Smlt3914 | 13.113 | 72.255 | Acetolactate synthase 2 catalytic subunit |
| Smlt3915 | 6.792 | 45.465 | Putative acetolactate synthase isozyme II small subunit |
| Smlt3916 | 5.969 | 81.936 | Threonine dehydratase |
| Smlt3917 | 17.588 | 53.598 | 2-isopropylmalate synthase |
| Smlt3918 | 6.752 | 66.989 | Putative trans-aconitate methyltransferase |
| Smlt3944 | 13.301 | 58.127 | Putative two-component regulatory system, sensor histidine kinase protein |
| Smlt3947 | 7.481 | 42.083 | Putative ABC transporter periplasmic solute-binding protein |
| Smlt3948 | 7.945 | 54.123 | Putative two-component system sensor histidine kinase protein |
| Smlt3950 | 2.687 | 38.97 | Putative phosphate selective porin |
| Smlt3951 | 5.944 | 43.627 | Citrate transporter |
| Smly3952 | 3.021 | 40.997 | 3-ketoacyl-(acyl-carrier-protein) reductase |
| Smlt3954 | 7.229 | 28.294 | Putative TonB dependent receptor protein |
| Smlt3970 | 3.404 | 29.974 | Putative type I HlyD transporter |
| Smlt3971 | 6.54 | 52.305 | Putative transmembrane transport protein |
| Smlt3973 | 2.651 | 64.078 | Putative universal stress-like protein |
| Smlt3975 | 12.242 | 45.018 | Putative GGDEF signaling/response regulator protein |
| Smlt3977 | 21.213 | 66.062 | Hypothetical protein |
| Smlt3981 | 14.192 | 42.612 | Putative NADH:flavin oxidoreductase family protein |
| Smlt3983 | 7.578 | 49.122 | Putative glyoxlase/bleomycin resistance protein |
| Smlt3984 | 4.351 | 21.2 | Putative hydrolase |
| Smlt3991 | 15.442 | 62.508 | Putative metallo-beta-lactamase superfamily protein |
| Smlt3998 | 13.357 | 57.864 | Endopeptidase |
| Smlt4016 | 4.088 | 23.826 | Hypothetical protein |
| Smlt4018 | 12.862 | 51.906 | Autotransporter protein |
| Smlt4019 | 21.821 | 84.521 | Transmembrane protein |
| Smlt4070 | 28.663 | 105.94 | Multidrug resistance outer membrane protein |
| Smlt4072 | 49.443 | 200.108 | Acriflavin resistance protein A |
| Smlt4104 | 8.711 | 47.705 | Hypothetical protein |
| Smlt4138 | 17.451 | 69.4 | Methyl-accepting chemotaxis protein |
| Smlt4140 | 6.454 | 25.528 | Hypothetical protein |
| Smlt4141 | 9.475 | 34.766 | Major facilitator superfamily transmembrane transporter |
| Smlt4144 | 8.151 | 39.678 | Hypothetical protein |
| Smlt4158 | 18.761 | 58.306 | Transmembrane DoxX family protein |
| Smlt4166 | 34.931 | 165.193 | D-tyrosyl-tRNA(Tyr) deacylase |
| Smlt4170 | 18.308 | 107.697 | Transmembrane protein |
| Smlt4180 | 26.263 | 110.035 | Smf protein |
| Smlt4201 | 14.311 | 97.489 | GIY-YIG nuclease superfamily protein |
| Smlt4210 | 4.791 | 43.806 | Hypothetical protein |
| Smlt4221 | 7.439 | 51.293 | Flavodoxin NAD-binding oxidoreductase |
| Smlt4222 | 20.775 | 63.78 | Methyl-accepting chemotaxis protein |
| Smlt4225 | 17.34 | 57.707 | Two-component sensor histidine kinase |
| Smlt4226 | 2.801 | 70.176 | Hypothetical protein |
| Smlt4227 | 8.035 | 33.261 | Carotenoid oxygenase |
| Smlt4228 | 9.549 | 45.032 | Hypothetical protein |
| Smlt4237 | 17.969 | 57.026 | Lyase/mutase |
| Smlt4238 | 3.502 | 39.705 | Hypothetical protein |
| Smlt4249 | 7.515 | 63.413 | Transmembrane protein |
| Smlt4250 | 13.048 | 57.995 | Transmembrane phosphatase |
| Smlt4270 | 2.526 | 40.38 | Hypothetical protein |
| Smlt4271 | 3.597 | 24.98 | Hypothetical protein |
| Smlt4272 | 5.469 | 42.542 | Hypothetical protein |
| Smlt4322 | 20.83 | 64.758 | Monovalent cation/H+ antiporter subunit D |
| Smlt4328 | 16.673 | 64.976 | Hypothetical protein |
| Smlt4333 | 27.202 | 87.73 | Transmembrane protein |
| Smlt4342 | 7.825 | 36.782 | Ehpr protein |
| Smlt4343 | 9.856 | 56.638 | DeoR family transcriptional regulator |
| Smlt4345 | 14.715 | 56.511 | GGDEF signalling protein |
| Smlt4348 | 22.817 | 75.947 | Phosphoglycerol transferase I |
| Smlt4354 | 3.376 | 38.193 | Oxidoreductase |
| Smlt4359 | 17.646 | 65.023 | Hypothetical protein |
| Smlt4361 | 10.09 | 39.136 | Two-component sensor histidine kinase |
| Smlt4362 | 1.337 | 4.112 | Hypothetical protein |
| Smlt4381 | 10.145 | 45.943 | Cold shock protein |
| Smlt4387 | 7.494 | 38.776 | TonB dependent receptor protein |
| Smlt4400 | 12.321 | 39.074 | Ubiquinol oxidase polypeptide I |
| Smlt4405 | 5.51 | 64.127 | GntR family regulatory protein |
| Smlt4410 | 5.808 | 40.527 | TonB dependent ferric enterobactin receptor |
| Smlt4412 | 7.315 | 48.43 | AraC family transcriptional regulator |
| Smlt4413 | 7.071 | 52.882 | Hypothetical protein |
| Smlt4415 | 19.45 | 73.382 | Transmembrane protein |
| Smlt4416 | 18.042 | 64.944 | Oligopeptidase |
| Smlt4429 | 17.896 | 56.179 | Transmembrane protein |
| Smlt4432 | 11.99 | 49.581 | TonB dependent receptor protein |
| Smlt4433 | 7.829 | 58.438 | Glycosyl hydrolase |
| smlt4434 | 10.488 | 70.64 | Sugar isomerase |
| Smlt4435 | 6.292 | 71.42 | ROK family protein |
| Smlt4436 | 11.638 | 52.403 | Tagatose 6-phosphate kinase |
| Smlt4439 | 16.293 | 63.09 | Major facilitator superfamily putative transmembrane transporter |
| Smlt4447C | 21.317 | 70.264 |  |
| Smlt4452 | 18.387 | 66.554 | Cell surface haemagluttinin protein |
| Smlt4454 | 5.534 | 62.896 | Hypothetical protein |
| Smlt4458 | 8.835 | 55.838 | Transmembrane chloride-channel protein |
| Smlt4459 | 8.197 | 53.541 | Biotin synthase |
| Smlt4461 | 14.854 | 91.693 | Biotin biosynthesis protein |
| Smlt4462 | 28.865 | 94.808 | Short chain dehydrogenase |
| Smlt4474 | 7.904 | 51.244 | Multidrug efflux system outer membrane protein |
| Smlt4475 | 8.778 | 35.104 | Multidrug efflux protein |
| Smlt4478 | 16.792 | 60.883 | Two-component system response regulator |
| Smlt4482 | 19.491 | 71.15 | Transmembrane protein |
| Smlt4483 | 17.766 | 69.416 | Transmembrane protein |
| Smlt4484 | 17.444 | 70.023 | Hypothetical protein |
| Smlt4486 | 14.291 | 53.722 | Hypothetical protein |
| Smlt4495A | 39.926 | 125.574 | Hypothetical protein |
| Smlt4506 | 30.394 | 100.183 | TonB dependent receptor protein |
| Smlt4508 | 9.57 | 74.615 | AraC family transcriptional regulator |
| Smlt4513 | 3.101 | 51.26 | GGDEF domain signalling protein |
| Smlt4535 | 19.576 | 85.756 | Transcriptional regulator |
| Smlt4537 | 17.205 | 71.706 | LysR family transcriptional regulator |
| Smlt4538 | 7.477 | 52.292 | Major facilitator superfamily transmembrane transporter |
| Smlt4558 | 14.726 | 92.507 | Dehydratase |
| Smlt4560 | 15.101 | 84.93 | Exported lipoprotein |
| Smlt4566 | 13.837 | 64.867 | Acyltransferase |
| Smlt4567 | 13.832 | 48.183 | Glycosyltransferase |
| Smlt4579 | 8.657 | 38.753 | RNA polymerase sigma factor |
| Smlt4580 | 12.767 | 61.547 | Transmembrane protein |
| Smlt4582 | 14.734 | 64.064 | NUDIX family protein |
| Smlt4584 | 13.847 | 66.219 | Hypothetical protein |
| Smlt1617 | 25.016 | 78.695 | 4'-phosphopantetheinyl transferase superfamily protein |
| Smlt4620 | 12.886 | 50.477 | Coniferyl aldehyde dehydrogenase |
| Smlt4630 | 0.09 | 0.357 | Transmembrane heat shock chaperone |
| Smlt4645 | 23.132 | 75.669 | Transmembrane methyl-accepting chemotaxis protein I |
| Smlt4651 | 7.427 | 31.514 | Hypothetical protein |
| Smlt4652 | 5.486 | 42.579 | Hypothetical protein |
| Smlt4653 | 19.553 | 68.933 | Transmembrane GGDEF signaling protein |
| Smlt4663 | 15.047 | 74.633 | Outer membrane efflux protein |
| Smlt4664 | 13.037 | 65.778 | Multidrug efflux protein |
| Smlt4667 | 37.518 | 119.789 | Exonuclease V |
| Smlt4674 | 39.805 | 133.223 | Anti-sigma B factor antagonist |
| Smlt4677 | 23.099 | 93.975 | Hypothetical protein |
| Smlt4680 | 8.882 | 67.508 | Dehydrogenase, zinc-containing |
| Smlt4681 | 13.494 | 53.731 | LysR family transcriptional regulator |
| Smlt4691 | 24.772 | 96.312 | tRNA modification GTPase TrmE |
|  |  |  |  |
| Genes significantly downregulated in the KJΔBC cells | | | |
| Smlt0341 | 356.107 | 59.122 | Putative arginase |
| Smlt0345 | 182.884 | 41.99 | Tryptophanyl-tRNA synthetase |
| Smlt0367 | 15.939 | 0.904 | Putative two-component response regulator transcriptional regulator |
| Smlt0381 | 167.656 | 37.521 | Putative protease |
| Smlt0383 | 275.994 | 48.02 | Putative histone H1-like protein |
| Smlt0387 | 6932.234 | 82.922 | Hypothetical protein |
| Smlt0390 | 202.898 | 44.921 | Hypothetical protein |
| Smlt0423 | 423.021 | 49.474 | Putative fatty acid transport system, membrane protein |
| Smlt0432 | 42.686 | 11.097 | Putative transmembrane protein |
| Smlt0444 | 1425.75 | 315.208 | 30S ribosomal protein S21 |
| Smlt0445 | 289.378 | 67.951 | Putative DNA-binding/iron metalloprotein/AP endonuclease |
| Smlt0483 | 128.503 | 42.319 | Hypothetical protein, YceI like family |
| Smlt0541 | 173.397 | 57.3 | Putative aminopeptidase |
| Smlt0557 | 73.47 | 11.757 | Hypothetical protein |
| Smlt0560 | 270.059 | 51.209 | Hypothetical protein |
| Smlt0569 | 330.327 | 63.619 | Putative transmembrane anchor protein |
| Smlt0598 | 20.56 | 6.808 | Putative O-antigen biosynthesis aminotransferase |
| Smlt0615 | 1866.073 | 305.529 | Hypothetical protein |
| Smlt0617 | 134.442 | 1.671 | Putative cystathionine beta/gamma-lyase |
| Smlt0656 | 381.472 | 120.06 | Putative succinyl-CoA:3-ketoacid-coenzyme A transferase subunit B |
| Smlt0657 | 120.258 | 37.789 | Putative glycosyltransferase protein |
| Smlt0669 | 284.17 | 94.285 | Putative transmembrane CDP-diacylglycerol--serine O-phosphatidyltransferase |
| Smlt0706 | 4820.966 | 969.168 | Putative fimbrial adhesin protein |
| Smlt0718 | 534.32 | 48.342 | Serine hydroxymethyltransferase |
| Smlt0723 | 104.386 | 0.405 | Putative deaminase |
| Smlt0748 | 402.737 | 76.276 | S-adenosyl-methyltransferase MraW |
| Smlt0764 | 362.329 | 96.682 | Preprotein translocase subunit SecA |
| Smlt0775 | 1240.063 | 88.762 | S-adenosyl-L-homocysteine hydrolase |
| Smlt0780 | 868.526 | 47.546 | S-adenosylmethionine synthetase |
| Smlt0794 | 158.521 | 26.338 | Hypothetical protein |
| Smlt0801 | 439.232 | 67.827 | Putative two-component system response regulator transcriptional regulator |
| Smlt0805 | 143.426 | 46.8 | Putative transmembrane protein |
| Smlt0841 | 1302.866 | 101.566 | Putative alkyl hydroperoxide reductase subunit c |
| Smlt0843 | 306.936 | 67.123 | Putative proline iminopeptidase |
| Smlt0862 | 200.747 | 44.432 | Putative asparaginase |
| Smlt0866 | 201.885 | 55.426 | Putative thioredoxin electron transport-like protein |
| Smlt0876 | 1393.124 | 149.008 | 50S ribosomal protein L25/general stress protein Ctc |
| Smlt1448 | 174.82 | 56.9 | Hypothetical protein |
| Smlt1657 | 2.13 | 0.562 | Hypothetical protein |
| Smlt2022 | 615.059 | 180.206 | Putative cold shock protein |
| Smlt2137 | 473.261 | 81.072 | Putative universal stress family protein |
| Smlt2144 | 19.622 | 0.399 | Putative two component system histidine kinase/response regulator fusion protein |
| Smlt3095 | 5.627 | 1.484 | Hypothetical protein |
| Smlt3409 | 493.942 | 73.276 | Putative flavonol synthase/dioxygenase |
| Smlt3419 | 201.387 | 63.924 | Tryptophan synthase subunit beta |
| Smlt3440 | 131.041 | 17.566 | Hypothetical protein |
| Smlt3774 | 81.226 | 19 | Sulfur carrier protein ThiS |
| Smlt4165 | 468.423 | 53.928 | RNA polymerase sigma factor RpoD |
| Smlt4298 | 339.818 | 69.437 | Bacterioferritin ferredoxin protein |
| Smlt4554 | 172.021 | 46.558 | Transmembrane protein |
