# Supplementary material for: Relationship of the CreBC two-component regulatory system and inner membrane protein CreD with swimming motility in Stenotrophomonas maltophilia
Source: PLoS One. 2017 Apr 24;12(4):e0174704. doi: 10.1371/journal.pone.0174704 (PMC5402928; doi:10.1371/journal.pone.0174704)
Supplement: S2 Table — (DOCX) [file pone.0174704.s003.docx]

**S2 Table. Bacterial strains, plasmids and primers used in this study**

| **Strain, plasmid, or primer** | **Genotype or properties** | **Reference** |
| --- | --- | --- |
| ***S. maltophilia*** |  |  |
| KJ | Wild type, a clinical isolate from Taiwan | 1 |
| KJΔCreB | *S. maltophilia* KJ mutant of *creB* ; *ΔcreB* | This study |
| KJΔCreC | *S. maltophilia* KJ mutant of *creC*; *ΔcreC* | This study |
| KJΔBC | *S. maltophilia* KJ mutant of *creBC* operon; *ΔcreBC* | 2 |
| KJΔBCD | *S. maltophilia* KJ mutant of *creBC* operon and *creD*; *ΔcreBC*, *ΔcreD* | This study |
|  |  |  |
| ***Escherichia coli*** |  |  |
| DH5α | F- φ80d/*acZΔM15* *Δ*(*lacZYA-argF*)*U169* *deoR recA1 endA1 hsdR17* (r_k_^-^ m_k_^+^) *phoA supE44λ* *thi-1 gyrA96 relA1* | Invitrogen |
| S17-1 | λ pir^+^ mating strain | 3 |
|  |  |  |
| **Plasmids** |  |  |
| pEX18Tc | *sacB oriT*, Tc^r^ | 4 |
| pRK415 | Mobilizable broad-host-range plasmid cloning vector, RK2 origin; Tc^r^ | 5 |
| pΔCreB | pEX18Tc with an internal-deletion *creB* gene; Tc^r^ | This study |
| pΔCreC | pEX18Tc with an internal-deletion *creC* gene; Tc^r^ | This study |
| pΔCreD | pEX18Tc vector containing the *creD* gene with an internal 519-bp deletion; Tc^r^ | 6 |
| pCreB(D55E) | pRK415 with an inserted intact *creB(D55E)* gene; Tc^r^ | 6 |
| pCreD | pRK415 with an intact *creD* gene; Tc^r^ | 6 |
| pCreB  pCreC | pRK415 with an intact *creB* gene; Tc^r^  pRK415 with an intact *creC* gene; Tc^r^ | This study  This study |
| **Primers** |  |  |
| CreB-F | 5’–CGGTCTAGAAGGCGTGGCGATAG– 3’ | This study |
| CreB-R | 5’–TGGGTACCCGGTAATCCAGCGAG –3’ |  |
| CreCN-F | 5’– TCAAGCTTTGGACCTGACCCG–3’ | This study |
| CreCN-R | 5’– GCCCAGGAACAGTCTAGAC–3’ |  |
| CreCC-F | 5’– CATCTAGACAACGTGCCCGAC– 3’ | This study |
| CreCC-R | 5’– CAGGAGCTCCAGCAGCAGCA–3’ |  |
| FliAQ-F | 5’–CGCCTCGTTCGAGACCTAT–3’ | This study |
| FliAQ-R | 5’–TTCCATCAACCGCAGGTATT–3’ |  |
| MotAQ-F | 5’–TGTGCTCAGCCTGCTCTATG–3’ | This study |
| MotAQ-R | 5’–TTTTCCAGCTCCAGTTCCAG–3’ |  |
| FliIQ-F | 5’–GGCTCGATCACTGCGTTC–3’ | This study |
| FliIQ-R | 5’–AGTAGGCCGAGACCAGTCG–3’ |  |
| FliQQ-F | 5’–CCCGAACTTGCCTTGACC–3’ | This study |
| FliQQ-R | 5’–GGAACAGCAGCGTGGTGAA–3’ |  |
| FliFQ-F | 5’–GTCAACGCTGCCCAGTTC–3’ | This study |
| FliFQ-R | 5’–GGTGGTGTTCTCGCTCATCT–3’ |  |
| FlgIQ-F | 5’–GCCGATGGCCAGATCTATG–3’ | This study |
| FlgIQ-R | 5’–GGTGGTGAAGTCGTTGTTGTGC–3’ |  |
| FlgEQ-F | 5’–ATTTCCGGTGAAGGCTTCTT–3’ | This study |
| FlgEQ-R | 5’–ACCTTCACTTCGCTGGTCTG–3’ |  |
| FliDQ-F | 5’–AATACCTCGGTGGGGAAGAC–3’ | This study |
| FliDQ-R | 5’–GCGATCGACAGGTACTGGTT–3’ |  |
| FliCQ-F | 5’–CCTGAGCTCGGTCAAGGA–3’ | This study |
| FliCQ-R | 5’–AGGGCCTTGTCGACCACT–3’ |  |

**References**

1. Hu RM, Huang KJ., Wu LT, Hsiao YJ, Yang TC. Induction of L1 and L2 β-lactamases of *Stenotrophomonas maltophilia*. Antimicrob Agents Chemother. 2008;52: 1198-1200.
2. Lin CW, Lin HC, Huang YW, Chung TC, Yang TC. Inactivation of *mrcA* gene derepresses the basal-level expression of L1 and L2 β-lactamases in *Stenotrophomonas maltophilia*. J Antimicrob Chemother. 2011;66: 2033-2037.
3. Simon R, O’Connell M, Labes M, Puhler A. Plasmid vectors for the genetic analysis and manipulation of rhizobia and other gram-negative bacteria. Methods enzymol. 1986;118: 640-659.
4. Hoang TT, Karkhoff-Schweizer RR, Kutchma AJ, Schweizer HP. A broad-host-range Flp-FRT recombination system for site-specific excision of chromosomally-located DNA sequences: application for isolation of unmarked *Pseudomonas aeruginosa* mutants. Gene. 1998;212: 77-86.
5. Keen NT, Tamaki S, Kobayashi D, Trollinger D. Improved broad-host-range plasmids for DNA cloning in gram-negative bacteria. Gene. 1988;70: 191-197.
6. Huang HH, Lin YT, Chen WC, Huang YW, Chen SJ, Yang TC. Expression and functions of CreD, an inner membrane protein in *Stenotrophomonas maltophilia*. PLoS One. 2015;10: e0145009.
